# Development of multi-epitope Cathepsin L driven short peptide vaccine against *Fasciola gigantica*

**DOI:** 10.3389/fvets.2025.1547937

**Published:** 2025-05-22

**Authors:** Supanan Chansap, Werachon Cheukamud, Thitikul Suthisintong, Pornanan Kueakhai, Narin Changklungmoa

**Affiliations:** Research Unit for Vaccine and Diagnosis of Parasitic Diseases, Faculty of Allied Health Sciences, Burapha University, Chonburi, Thailand

**Keywords:** Cathepsin L, *Fasciola gigantica*, immunoinformatic, multi-epitope, peptide vaccine

## Abstract

Fasciolosis is an important zoonotic disease caused by *Fasciola* species (*Fasciola* spp.). *Fasciola* spp. infection has the potential to affect the livestock economy. Furthermore, liver flukes have been found to present Triclabendazole resistance in many countries. Vaccines are used to prevent fasciolosis and are currently considered the best alternative. However, no liver fluke vaccine is commercially available at present. *Fasciola gigantica* Cathepsin Ls (FgCatLs) are vital enzymes for the liver fluke’s survival. Therefore, this study aimed to design and investigate the immune response of multi-epitope Cathepsin L (MeCatL) driven short peptide vaccine for fasciolosis using immunoinformatic tools. FgCatLs sequences were predicted Linear B cell (BCL)- and Helper T lymphocyte (HTL)-specific immunogenic Eepitopes. The selected epitopes were marked on FgCatL’s alignments. Novel epitopes were constructed from three criteria, including the selection process taking non-conserved host regions, overlapping FgCatLs sequences, and the highest percent conserved residues. Novel epitopes of BCL and HTL were linked with a linker to design a short peptide. MeCatL driven short peptide presented high antigenicity, non-allergenicity, non-toxicity, and good solubility. MeCatL driven short peptide was predicted and refined the tertiary structure. The refined MeCatL driven short peptide model indicated good quality structure that was investigated by Ramachandran plot, ERRAT, and Z-score. The refined MeCatL driven short peptide model interacted with Toll-like receptor 2 (TLR-2). The lowest energy was −1222.4 kJ/mol. The levels of IgM, IgG1, and IgG2 were increased in *in silico* immune simulation. MeCatL driven short peptide was synthesized and immunized in mice. IgG1 and IgG2a levels were increased after week 2, indicating IgG1 levels were dominating. MeCatL driven short peptide immunized sera can detect single proteins, including rFgCatL1, rFgCatL1G, and rFgCatL1H. In addition, MeCatL driven short peptide immunized sera was specifically detected in the cecal epithelium of NEJ and adult stages. These findings suggest that the MeCatL short peptide is a promising vaccine candidate, capable of inducing targeted immune responses, though further studies are needed to confirm its protective efficacy *in vivo*.

## 1 Introduction

Fasciolosis is a ruminant disease caused by *Fasciola* spp., including *Fasciola gigantica* (*F. gigantica*) and *Fasciola hepatica* (*F. hepatica*). *Fasciola gigantica* is found in subtropical and tropical regions, but *F. hepatica* is commonly found in the temperate region of the world (1). *Fasciola* spp. affects the agricultural economy through milk and meat production losses, weight loss, reduced fertility, health span of animals, and increased animal mortality (2–4). The incidence of human infections has been increasing worldwide, especially in developing and under-developing countries (1, 5). Additionally, the World Health Organization (WHO) has reported that at least 2.4 million humans have fasciolosis in 70 countries around the world (6); therefore, fasciolosis is considered a severe public health concern worldwide (7–9). Liver flukes have several stages after entering the host, each of which is associated with different levels of protein expression. The Newly excyst juvenile (NEJ) stage is an important stage after the metacercariae enter the host, being the first stage after liver flukes excyst from the metacercariae. Therefore, the protein secretion characterizing the NEJ stage can be used to determine candidates for vaccination. According to previous studies, several proteins have been investigated for their potential as vaccines against fasciolosis, including Fatty acid-binding protein (FABP) (10), Saponin-like protein-1 (SAP-1) (11), Saponin-like protein-2 (SAP-2) (12), Glutathione S-transferase (GST) (13, 14), Leucine aminopeptidase (LAP) (15), Hemoglobin (16), Peroxiredoxin (Prx) (17, 18), Superoxide dismutase (SOD) (19), Cathepsin L1 (CatL1) (20–22), Cathepsin L1H (CatL1H) (23), Cathepsin L1G (CatL1G) (24), Cathepsin B2 (CatB2), and Cathepsin B3 (CatB3) (7). As mentioned above, Cathepsins are major proteins in all liver fluke stages (25, 26).

*Fasciola gigantica* Cathepsin Ls (FgCatLs) are essential enzymes, which are highly expressed in all stages by liver flukes (27). FgCatLs have many isotypes, such as *Fasciola gigantica* Cathepsin L1 (FgCatL1), *F. gigantica* Cathepsin L1B (FgCatL1B), *F. gigantica* Cathepsin L1C (FgCatL1C), *F. gigantica* Cathepsin L1D (FgCatL1D), *F. gigantica* Cathepsin L1E (FgCatL1E), *F. gigantica* Cathepsin L1F (FgCatL1F), *F. gigantica* Cathepsin L1G (FgCatL1G), and *F. gigantica* Cathepsin L1H (FgCatL1H). Furthermore, FgCatLs play crucial roles regarding survival in the host. Many studies have demonstrated the involvement of these proteins in parasite invasion, digestion, immune evasion, and excystment (28–30). Moreover, FgCatLs have been observed as different isotypes in each parasite stage. FgCatL1, FgCatL1B, FgCatL1C, and FgCatL1D were mainly found to be expressed in the adult stage (31, 32), while FgCatL1G was observed in the NEJ and juvenile stages (24, 31). FgCatL1H were mainly found in the juvenile stage (23). In addition, FgCatL1E and FgCatL1F were found in a partial protein sequence from *F. gigantica* (31). Previous studies, FgCatL1, FgCatL1G, and FgCatL1H showed a high percentage of protection compared with control groups (23).

The host’s immunological response follows the entry of the liver flukes into the host’s body, where the immune responses directed against liver flukes involve both humoral and cellular immunity pathways (30, 33, 34). The humoral immune response plays a main role against parasite infection through the production of antibodies. In addition, the cellular immune response can recognize parasite antigens via Antigen Presenting Cells (APCs) such as macrophages, dendritic cells, and Langerhans cells. APCs present antigens through the major histocompatibility complex (MHC) class II on T helper (Th) 2 cells. Th2 cells secrete cytokines that stimulate the production of immunoglobulin by B-cells, activate effector cells, enhance smooth muscle contractility, increase mucus production by goblet cells, and induce epithelial cell permeability (30, 35). In addition, Antibody-Dependent Cellular Cytotoxicity (ADCC) is an important mechanism of the humoral immune response against extracellular parasites. ADCC is a process that depends on a specific antibody that attaches to a parasite antigen and specifically stimulates effector cells, natural killer (NK) cells attach to the Fc receptor of the antibody. This interaction triggers degranulation, leading to destruction of the parasite antigens (33, 36).

At present, fasciolosis treatment is based on Triclabendazole, the most effective drug compared to other drugs. However, it has been reported that liver flukes are resistant to Triclabendazole in many countries, including Turkey, Netherlands, Peru, and Chile (4, 37–40). Furthermore, fasciolosis vaccines are not yet commercially available. Vaccines are considered an attractive alternative for the prevention of fasciolosis, as they are more sustainable, safe, environmentally friendly, and cost-effective (4, 34, 41). As mentioned previously, several vaccines against *Fasciola* spp. employ recombinant proteins for vaccination, which have the ability to stimulate the immune response in animals and protect them from *F. gigantica* infection. Prior vaccine trials have mostly emphasized the synthesized full-length and mature proteins expressed by bacteria or yeast expression systems. The advantages of bacteria and yeast expression systems are their fast growth rate and high protein yield. However, bacteria expression systems face problems including the inability to conduct post-translational modification, different codon usage, contamination from bacteria, and the production of insoluble proteins. In addition, yeast expression systems are time-consuming, involve complex propagation and high-cost purification, and carry the risk of yeast contamination (42). The aforementioned issues with recombinant protein expression make peptide vaccines an attractive alternative for fasciolosis vaccine development.

Peptide vaccines, also known as epitope vaccines, are a type of subunit vaccine that mimic specific regions of antigens (called epitopes) and stimulate strong and immediate immune responses (43, 44). T lymphocytes (T cells) recognize free antigens through the MHC on the surface of APCs, while B cells recognize free antigens via secreted antibodies or B cell receptors (45–47). The advantages of peptide vaccines are their low cost, increased stability, unlimited synthesis, ease of production, short production time, safety, less likely to cause an allergic reaction or autoimmune response, no biological contamination (48, 49). Furthermore, peptide vaccines can be specifically engineered to elicit the intended immune response, a feat that is unattainable with conventional vaccines such as live attenuated vaccine, inactivated vaccine, recombinant protein vaccine, and toxoid vaccine (50). The desired immune response of peptide vaccines can assist in overcoming Human Leukocyte Antigens (HLA) polymorphisms (47, 51).

*In-silico* computer-based techniques have been used to improve the efficiency of peptide vaccines (52). Immunoinformatic tools can accurately predict the epitopes of B cells and T cells, allowing for the efficient development of pathogen-specific memory, which is closely linked to adaptive immunity (53, 54). In recent years, many studies have used immunoinformatic tools to develop and test vaccines against various parasites, including *Fasciola hepatica* (51), *Schistosoma mansoni* (55, 56), *Trichinella spiralis* (57), *Trichuris trichiura* (58), and *Ascaris suum* (59). In addition, those studies used the epitopes of each protein to connect to multiple proteins. Therefore, this study aims to predict the Linear B cell (BCL)- and Helper T lymphocyte (HTL) immunogenic epitopes from FgCatLs using immunoinformatic tools. After that, novel epitopes are selected and generated to design the multi-epitope Cathepsin L (MeCatL) driven short peptide vaccine. In addition, the MeCatL driven short peptide was also investigated for immune response *in silico* immune simulation and mice experiments.

## 2 Materials and methods

### 2.1 Amino acid sequence retrieval

The amino acid sequences of FgCatLs, including FgCatL1 (AAD23996), FgCatL1B (AAF44675), FgCatL1C (AAF44676), FgCatL1D (AAF44677), FgCatL1E (AAF44678), FgCatL1F (AAF44679), FgCatL1G (AAL23917), FgCatL1H (AAR08900), *Homo sapiens* Cathepsin L (HsCatL, NP_666023), *Bos taurus* Cathepsin L (BtCatL, CAA62870), *Mus musculus* Cathepsin L (MmCatL, NP_034114), and *Capra hircus* Cathepsin L (ChCatL, AHL24624) were obtained in FASTA format from the GenBank database.^1^ Moreover, FgCatLs were predicted for physicochemical properties, allergenicity, antigenicity, and solubility using Expasy,^2^ AllerTop v 2.0,^3^ Vaxijen v 2.0,^4^ and PepCalc server,^5^ respectively.

### 2.2 Linear B-cell epitope prediction

Protein sequences were selected from the mature protein sequence, which predicted Linear B-cell (BCL) epitopes by the Immune Epitope Database (IEDB) server^6^ using Bepipred Linear Epitope Prediction 2.0 based on the epitope/non-epitope predictions. The scores predicted an epitope (default value is 0.5) (60). The selected epitopes were evaluated for the antigenicity using Vaxijen v 2.0. Epitopes with a score over 0.5 were chosen as epitopes in the server’s output.

### 2.3 Helper T lymphocyte epitope prediction

Helper T lymphocyte (HTL) epitopes were predicted by the IEDB server.^7^ Each protein sequence was submitted with the following the IEDB recommended 2023.05 (NetMHCIIpan 4.1 EL), the selected species/locus was a mouse (H-2-I), the selected allele was H2-IAb and H2-IAd, and the selected length was 15 aa (default). The output of prediction percentile ranks. The percentile rank was a transformation that normalized the prediction scores across different MHC molecules and enabled a specific comparison of MHC-binding predictions (61). The 10 percentile rank values were selected for the epitopes. In addition, antigenic values were predicted using the Vaxijen v 2.0. In the output of this server, epitopes with a value greater than 0.5 score were selected as epitopes.

### 2.4 Selection and construction of the novel BCL and HTL epitopes

All FgCatLs sequences were aligned with the host’s Cathepsin L, including HsCatL, BtCatL, MmCatL, and ChCatL using the Clustal Omega server.^8^ All FgCatLs alignments were used to find conserved residues of FgCatLs proteins. All FgCatLs with host’s Cathepsin L alignments were used to find the Cathepsin L conserved sequence. All selected BCL and HTL epitopes of FgCatLs were labeled in the FgCatLs alignment. The novel BCL and HTL epitopes were constructed using 3 criteria, including (1) a non-conserved host region, (2) the epitopes were selected from overlapping amino acid sequences, and (3) the highest percent FgCatLs conserved residue. Subsequently, the amino acid sequence of overlapped BCL and HTL epitopes was used to construct the novel BCL and HTL epitopes. The novel BCL and HTL epitopes represent selected all FgCatLs epitopes.

### 2.5 Design of the MeCatL driven short peptide

**Figure 1:**
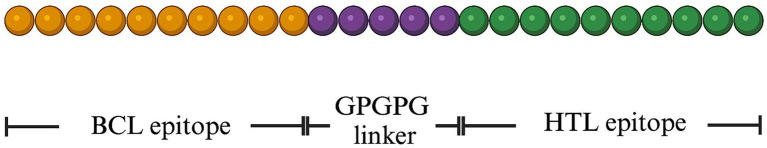
Schematic representation of the final MeCatL driven short peptide structure. The peptide was 40 amino acid (aa) in length. Yellow, violet, and green colors indicate the novel BCL epitope, GPGPG linker, and the novel HTL epitope, respectively.

After construction of novel BCL and HTL epitopes, the MeCatL driven short peptide divides into 3 parts, including the novel BCL epitope, the linker, and the novel HTL epitope. The novel BCL epitope sequence was located on N-terminal side. After that, the linker (GPGPG linker) was connected the novel BCL epitope sequence, which used to separate novel BCL and HTL epitopes. The HTL epitope sequence was connected with the GPGPG linker sequence. The HTL epitope sequence was located on the C-terminal side (Figure 1).

### 2.6 Percent identity of the MeCatL driven short peptide

The percent identity of the MeCatL driven short peptide was aligned with the mature sequence of the host’s Cathepsin L, including HsCatL, BtCatL, MmCatL, ChCatL. In addition, the MeCatL driven short peptide was aligned with the mature sequence of FgCatLs protein, including FgCatL1, FgCatL1B, FgCatL1C, FgCatL1D, FgCatL1E, FgCatL1F, FgCatL1G, and FgCatL1H. The percent identity was validated using the Clustal Omega v 1.2.4 server (see text footnote 8).

### 2.7 Prediction of physicochemical properties, antigenicity, allergenicity, toxicity, and solubility

The short peptide sequence driven by MeCatL was predicted based on its physicochemical properties using tools available on the ExPASy server. Antigenicity was predicted using the Vaxijen v 2.0 server. Vaxijen was based on the physicochemical properties of proteins without recourse to sequence alignment (62), allergenicity using the AllerTop v 2.0 server, which was based on a training set containing 2,427 known allergens from different species and 2,427 non-allergens (63), toxicity using the ToxinPred 2.0 server. ToxinPred was based on toxic/non-toxic peptides (64), and solubility using the PepCalc server, which was based on the iso-electric point, the peptide length, and the number of charged residues (65).

### 2.8 Prediction, refinement, and validation of tertiary structure

The MeCatL driven short peptide sequence was predicted as the tertiary structure using the AlphaFold2 server.^9^ AlphaFold2 server was a deep learning prediction of protein structure, which was based on multiple sequence alignment (MSA) features (66, 67). The model with the highest predicted local distance difference test (pLDDT) score was selected for refinement by the GalaxyRefine server.^10^ The pLDDT scores were in the range of the residue structure confidence. GalaxyRefine server refined loop and terminus regions using *ab initio* modeling (68). After that, Pymol software v 2.5.4 was used to visualize models. In addition, the vaccine model was validated by Ramachandran plot, ERRAT using SAVES v6.0 server.^11^ In addition, the Z-score was calculated by ProSA-web^12^ (69).

### 2.9 Prediction of discontinuous and continuous B-cell epitopes

Discontinuous and continuous B-cell epitopes were predicted using the ElliPro server^13^ for further analysis of MeCatL driven short peptide. ElliPro was based on a protein antigen’s 3D structure, residue protrusion index, and neighboring residue, which predicted linear and discontinuous BCL epitopes (70). The server used default parameters, including 6 Å maximum distance and 0.5 minimum scores.

### 2.10 Molecular docking and molecular dynamic simulation of MeCatL driven short peptide candidate with Toll-like receptor 2

Molecular docking was performed on the binding interactions between the receptor and ligand of the MeCatL driven short peptide. The refined peptide model was docked to Toll-like receptor 2 (TLR-2) (PDB ID: 5D3I) by the ClusPro2.0 server,^14^ which was based on fast Fourier transform (FFT) algorithms. FFT-based methods were used in hundreds of thousands of docking calculations (71), a docking complex was selected for analysis. After that, Pymol software v 2.5.4 was used to visualize the molecular docking.

MD simulation of the docking complex was further analyzed using the iMOD server (IMODS)^15^ with 9,000 cycles, 300 K constant temperature, and a constant pressure of 1 atm for 50 ns molecular dynamic simulation. IMODS was used for a rapid molecular dynamic simulation study that was performed (72, 73). The docked model with the lowest binding affinity was used to upload PDB files to the server, with all parameters set to default. The stability of the docked model was described as deformability, B-factors, eigenvalues, variance, covariance maps, and elastic network model.

### 2.11 *In-silico* immune response simulation

Immune response simulation was predicted using the C-immSim server.^16^ C-immSim server that predicted cellular and humoral immune response after vaccination. This server used machine learning algorithms and site-specific scoring matrices (PSSM) to predict epitopes and assess immunological interactions (74). The three doses were administered at the suggested 4-week intervals and at time steps 1, 84, and 168 (one time step is 8 h of real life). Simulation steps were adjusted to 1,050. All parameters were kept for the default simulation parameters (75).

### 2.12 Production of recombinant proteins

The recombinant *F. gigantica* Cathepsin L1 (rFgCatL1), recombinant *F. gigantica* Cathepsin L1G (rFgCatL1G), and recombinant *F. gigantica* Cathepsin L1H (rFgCatL1H) were expressed in *E. coli* BL21(DE) and purified as previously studies (22, 24, 76). Briefly, the single colony was picked up and inoculated in 100 mL LB Broth incubated at 37°C overnight with a shaking incubator. The incubated culture was inoculated with 40 mL in 4 L of LB Broth containing 100 μg/mL of kanamycin (Gibco, Thermo Fisher Scientific, Waltham, MA, United States, Cat. No. 15160054). This culture was incubated 37°C with shaking incubator. The 1 mM isopropyl-*β*-D-thiogalactoside (IPTG) (Sigma-Aldrich, St. Louis, MO, United States, Cat. No. I6758) was added when the culture grew at OD600 as 0.6. This culture was incubated for 3 h (hr) at 37°C. Finally, the culture was centrifuged for 30 min (min) at 4,000 g. The pellet was collected. The pellet was used to perform the protein purification by nickel-nitrilotriacetic acid (Ni-NTA) affinity-chromatography (QIAGEN, Hilden, Germany, Cat. No. 30210). The rFgCatL1 and rFgCatL1G were eluted by denaturing conditions. The elutes were dialyzed using SnakeSkin™ Pleated Dialysis Tubing (Thermo Scientific, Waltham, MA, United States, Cat. No. 68100). The Amicon Ultra centrifugal filter devices (Millipore, Bedford, MA, United States, Cat. No. UFC901024) were used for protein concentrations. The rFgCatL1 and rFgCatL1G protein concentrations were determined by Lowry’s method (77).

### 2.13 MeFgCatL peptide synthesis and antibody production in mice

**Figure 2:**
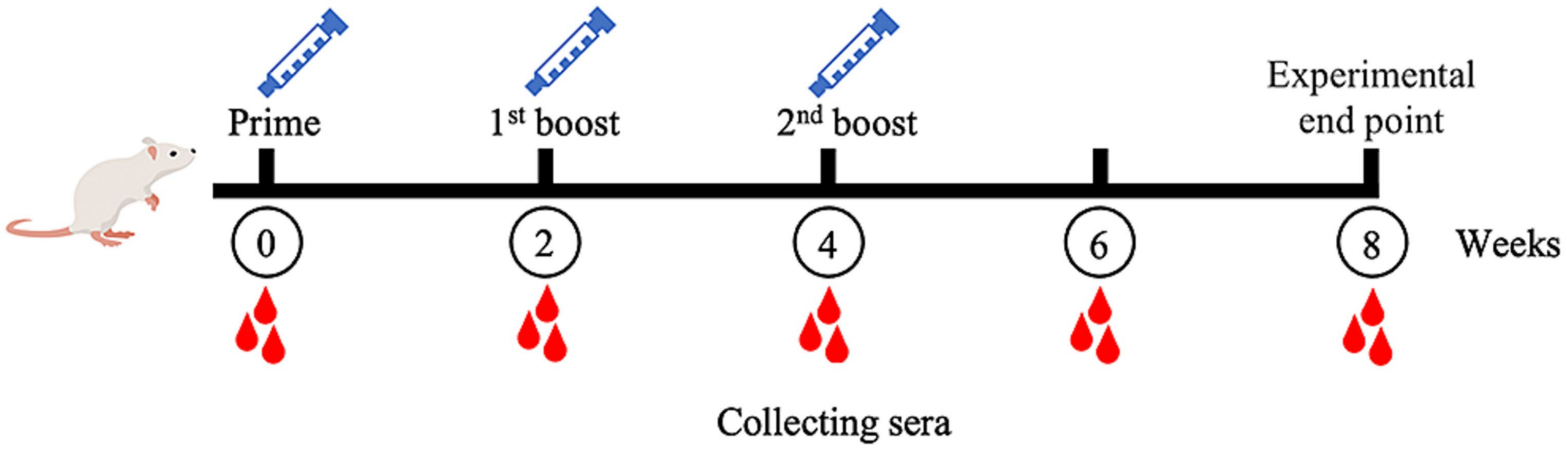
Schematic of mice experimental protocol. Mice immunizations were injected with MeCatL driven short peptide in Quil-A adjuvant at four times, two-week intervals (prime, 1st boost, 2nd boost). Blood sera were collected at the time point. Week 8 was the termination for further analysis.

The MeCatL driven short peptide was synthesized by the GenScript company. In the mouse experiment, five 8-week-old male ICR mice were used. The experiment was approved and managed according to protocol by The Animal Care and Use Committee of Burapha University, Thailand (Protocol code: IACUC 025/2565 and date of approval: 16th of September 2022). For immunization, Prime dose used 50 μg of the MeCatL, followed by 2 boosts (first and second boost) of 25 μg of the MeCatL each. MeCatL were mixed with Quil-A adjuvant (Invitrogen, Carlsbad, CA, United States, Cat. No. 1865007) and immunized via subcutaneous injection to individual mice. Mice were immunized three times, spaced 2 weeks apart. Mouse sera was taken 2 weeks prior to each immunization. Week 8 was the experimental endpoint (Figure 2). Blood was collected through the saphenous vein at weeks 0, 2, 4, 6, and 8. The cut-off value was determined by the mean OD from pre-immunized sera.

### 2.14 Determination of IgG1 and IgG2a levels

Using indirect ELISA, IgG1 and IgG2a levels in mouse sera were determined and triplicates were performed. In the MeCatL driven short peptide, U96 Maxi Sorp Nunc-Immuno Plate (Thermo Fisher Scientific, Waltham, MA, United States, Cat. No. 446261) were coated with 50 μL of 2 μg/mL of the MeCatL driven short peptide in coating buffer pH 9.6 (35 mM NaHCO_3_, and 15 mM Na_2_CO_3_) overnight at 4°C. The coated plates were washed three times with 0.05% PBST. Block nonspecific binding with 100 μL per well of 1% bovine serum albumin (BSA) (Capricorn Science, Ebsdorfergrund, Germany, Cat. No. BSA-1000) in PBS was added and incubated at room temperature (RT) for 1 h. The plates were then washed three times with 0.05% PBST, loaded with mouse sera diluted with PBS at 1:50 and incubated for 2 h at RT. After incubating, plates were washed three times with 0.05% PBST and added horseradish peroxidase (HRP)-conjugated with goat anti-mouse IgG1 or IgG2a (Southern Biotech, Birmingham, AL, United States, Cat. No. 5300–05) diluted with PBS at 1:500 to incubated for 2 h at RT. The plates were washed with 0.05% PBST five times, 50 μL of 3, 3′, 5, 5′-Tetramethylbenzidine (TMB) (Sigma, St. Louis, MO, United States; Cat. No. T0440) was added and incubated for 15 min at RT. 50 μL of stop buffer (1 N HCl) was added to each well to stop the reaction. The optical densities (OD) in an automated VersaMax Microplate Reader (Molecular Devices, CA, United States) were measured at 450 nm. In addition, MeCatL driven short peptide, rFgCatL1, rFgCatL1G, and rFgCatL1H proteins were determined IgG1 and IgG2a levels with week 8 according to previous protocol.

### 2.15 Parasite tissues preparation

The fresh *F. gigantica* were preserved with 4% paraformaldehyde in PBS (140 mM NaCl, 2.7 mM KCl, 10 mM Na_2_HPO_4_, pH 7.4) at 4°C for 4 h. After that, the parasite tissues were dehydrated with a graded series of ethyl alcohol (70, 80, 90, 95, and 100% concentrations, three times for 1 h each). The parasite tissues were cleared with xylene three times for 1 h. Finally, parasite tissues were infiltrated with paraplast at 60°C two times for 1 h. The parasite tissues were embedded in paraffin and cut into sections 5 μm in thickness by microtome. The sections were placed on the silane coated slides (3-aminopropyl-triethoxysilane) (Sigma-Aldrich, St. Louis, MO, United States, Cat. No. A3648) and dried immediately on a hot plate at 40°C overnight. Finally, the sections were kept for the further experiment.

### 2.16 Immunolocalization of *Fasciola gigantica*

The parasite tissues were dewaxed with xylene and rehydrated with 100, 95, 80, and 70% ethyl alcohol for 5 min at a time. The tissue sections were microwaved at 700 watts in citrate buffer (10 mM citric acid, pH 6.0) for 5 min at three times. The sections were washed with tap water for 5 min and followed by 0.1% PBST. The sections were blocked nonspecifically by 4% BSA in PBS for 1 h. After that, the MeCatL driven short peptide immunized sera diluted with PBS containing 1% BSA were added to slides and incubated overnight at 4°C. The sections were washed with 0.1% PBST. The sections were added alkaline phosphatase (AP)-conjugated with goat anti-mouse IgG (Invitrogen, Carlsbad, CA, United States, Cat. No. 31320) for 1 h at RT and washed with 0.1% PBST. After that, the slides were developed with the substrates nitrobluetetrazolium chloride/5-bromo-4-chloro-3-indodyl phosphate (NBT/BCIP) (Merck, Darmstadt, Germany, Cat. No. 11681451001) in the dark. The optimal level of color development was stopped by stop buffer (TBS, 20 mM EDTA, pH 8.0). The slides were mounted with 90% glycerol and visualized by light microscope.

## 3 Results

### 3.1 The primary analysis of amino acid sequence retrieval

**Figure 3:**
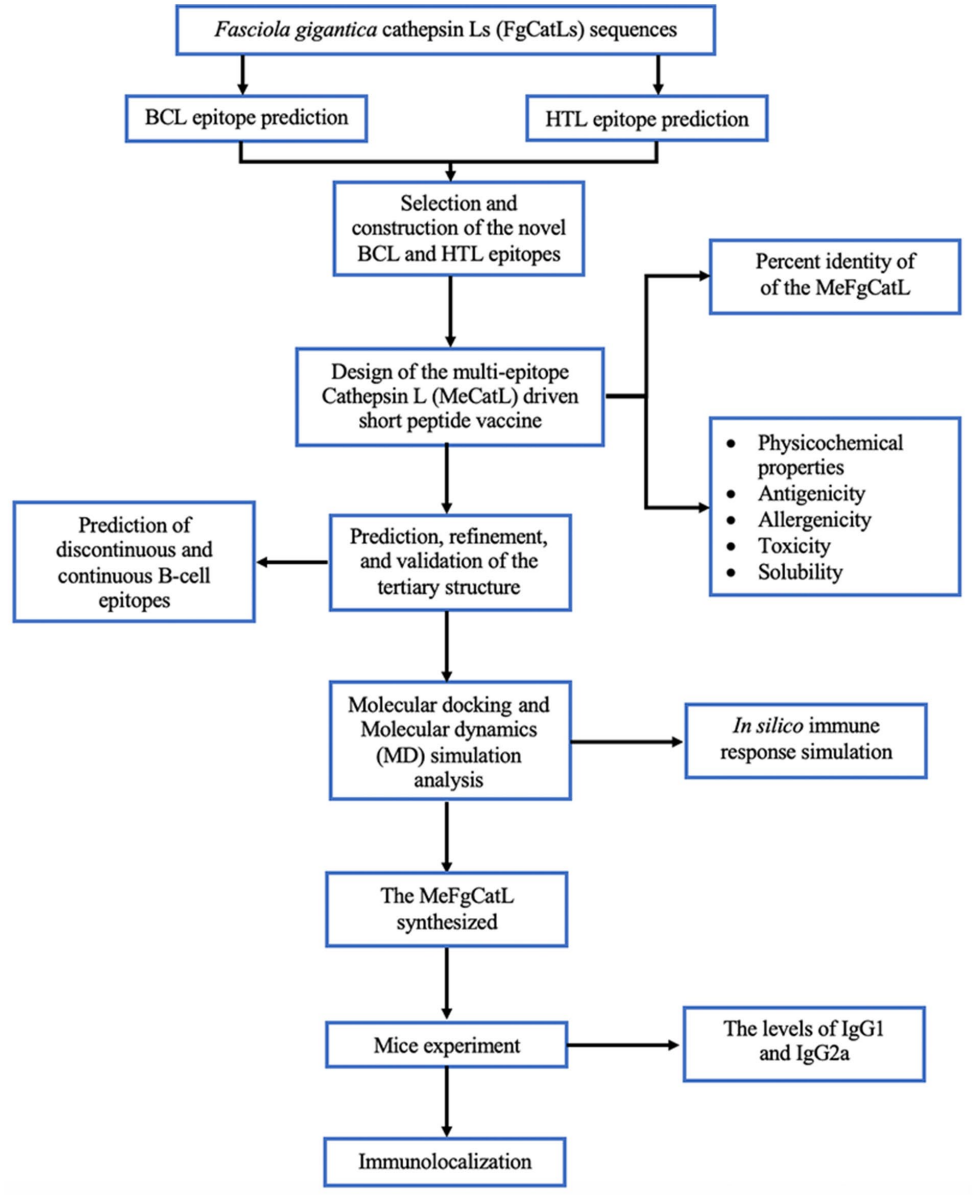
Flowchart summarizing the step of the design and determination of MeCatL driven short peptide.

FgCatLs sequences were retrieved in FASTA format from the GenBank database. The physicochemical properties, allergenicity, antigenicity, and solubility of FgCatL’s mature sequences were calculated using Expasy, AllerTop v 2.0, Vaxijen v 2.0, and PepCalc server, respectively. According to the results shown in Table 1, antigenicity ranges from 0.3459 to 0.5652, molecular weight ranges from 24.097 to 24.868 kilodaltons (kDa), and the grand average of hydropathicity (GRAVY) scores ranges from −0.426 to −0.305. In addition, all FgCatLs sequences were predicted to be non-allergen except FgCatL1 and FgCatL1H. FgCatL1H was predicted to have good solubility. All FgCatL’s mature sequences were predicted using immunoinformatic tools, which were analyzed to determine BCL and HTL epitopes. In addition, antigenicity, allergenicity, toxicity, and solubility were used to validate the properties of the MeCatL driven short peptide. Prediction, refinement, and validation of tertiary structure were constructed. Discontinuous and continuous B-cell epitopes were predicted. The interaction protein was used to dock between MeCatL driven short peptide and TLR-2. The MD simulation analysis was used to investigate the docked model. The whole flowchart represents the overall procedure of the MeCatL driven short peptide design and determination in Figure 3.

**Table 1:** Properties of mature FgCatLs.

Protein	Accession ID	Antigenicity	Allergenicity	Solubility	Molecular weight (kDa)	GRAVY
FgCatL1	AAD23996	0.5343	Yes	Poor	24.097	−0.365
FgCatL1B	AAF44675	0.5407	No	Poor	24.129	−0.378
FgCatL1C	AAF44676	0.4396	No	Poor	24.238	−0.324
FgCatL1D	AAF44677	0.5464	No	Poor	24.450	−0.474
FgCatL1E	AAF44678	0.5272	No	Poor	24.217	−0.380
FgCatL1F	AAF44679	0.3459	No	Poor	24.415	−0.305
FgCatL1G	AAR08900	0.5652	No	Poor	24.868	−0.426
FgCatL1H	AAL23917	0.5209	Yes	Good	24.267	−0.388

### 3.2 BCL epitope prediction

**Figure 4:**
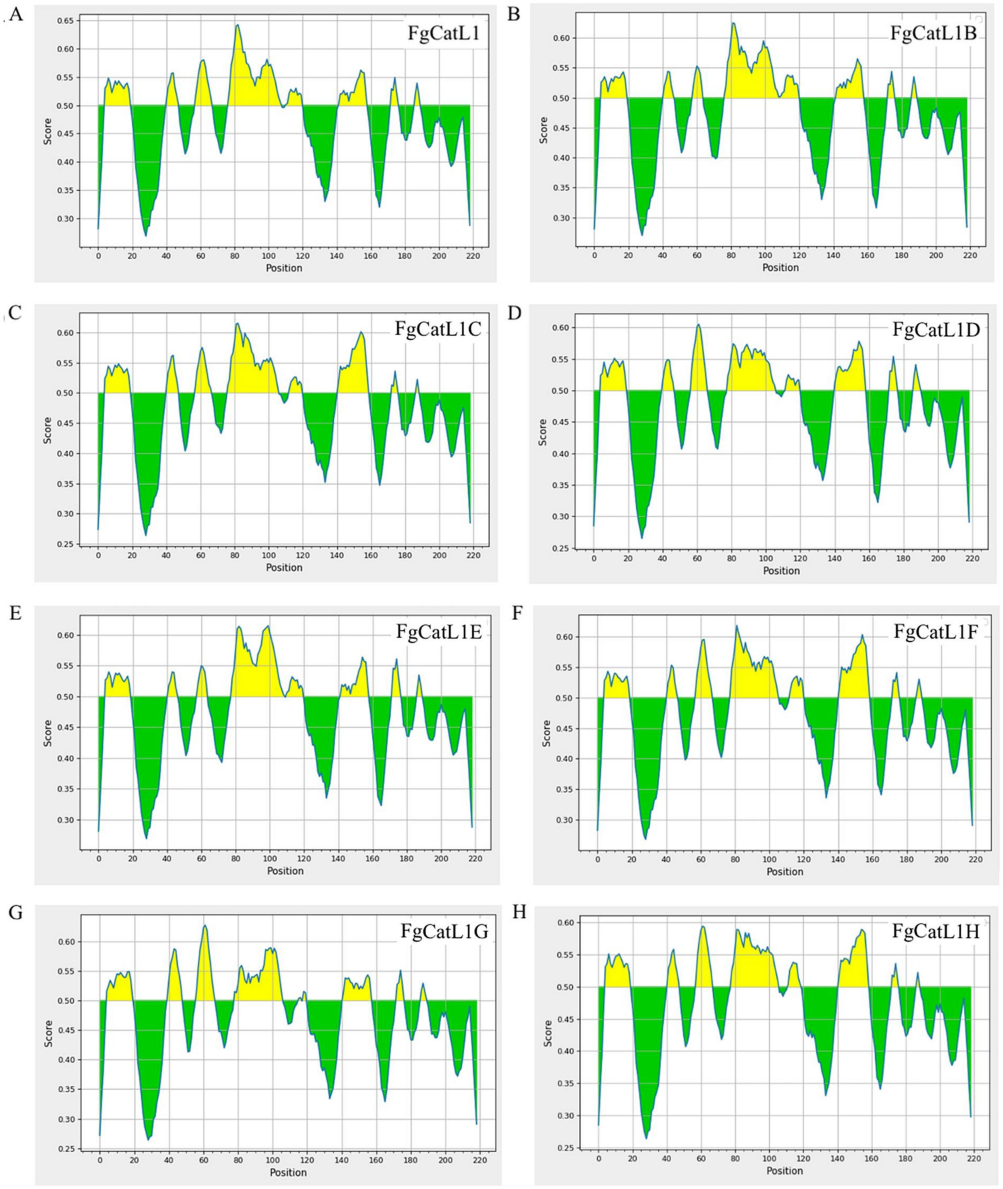
BCL epitopes of *Fasciola gigantica* sequences. **(A)** FgCatL1, **(B)** FgCatL1B, **(C)** FgCatL1C, **(D)** FgCatL1D, **(E)** FgCatL1E, **(F)** FgCatL1F, **(G)** FgCatL1G, and **(H)** FgCatL1H are linear B-cell epitopes. The score cut-off and yellow highlighted regions above the threshold line (default value is 0.5) are BCL epitopes (where Y-axes depict residue scores and X-axes residue positions in the sequence).

BCL epitopes were predicted by the IEDB server using Bepipred Linear Epitope Prediction 2.0, which selected a threshold score cut-off of more than 0.5. The yellow highlighted regions above the threshold line indicated peptide sequences can be an epitope for B-cells. The green highlighted regions under the threshold line indicated non-epitope for B-cells. These results were indicated in Figures 4A–H. In addition, the BCL epitopes were selected from antigenic score more than 0.5, as indicated in Table 2. The total BCL epitopes of selected FgCatL1, FgCatL1B, FgCatL1C, FgCatL1D, FgCatL1E, FgCatL1F, FgCatL1G, and FgCatL1H have 2, 4, 3, 4, 2, 3, 4, and 2 epitopes, respectively. The length of the BCL epitope ranges from 6 to 28 amino acids (aa). The antigenic score of selected BCL epitope results ranges from 0.5459 to 3.3362.

**Table 2:** Selected BCL epitopes for mature FgCatLs sequences.

No.	Start	Stop	Peptide sequences	Lengths (aa)	Antigenicity
FgCatL1					
1	5	19	IDWRESGYVTELKDQ	15	1.5126
2	57	66	GPWGNMGCSG	10	3.2924
FgCatL1B					
1	5	20	IDWRESGYVTEVKDQG	16	1.6561
2	41	47	NERTSIS	7	0.9697
3	59	64	PWGNYG	6	1.2629
4	143	158	TMYSGGIYQSRTCSSL	16	0.9125
FgCatL1C					
1	5	19	IDWRESGYVTEVKDQ	15	1.5650
2	58	67	DDFGNFGCNG	10	2.7452
3	113	120	MVHSGDEV	8	0.5459
FgCatL1D					
1	5	20	IDWRDYYYVTEVKDQG	16	1.1810
2	41	47	NERASAS	7	1.4420
3	58	66	RNFGNHGCG	9	1.9198
4	79	106	HSGLETDSYYPYQAVEGPCQYDGRLAYA	28	0.7485
FgCatL1E					
1	5	19	IDWRESGYVTEVKDQ	15	1.5650
2	59	64	PWGNYG	6	1.2629
FgCatL1F					
1	5	19	IDWRDSGYVTKVKDQ	15	1.3030
2	58	67	SDFGNNGCRG	10	2.8975
3	113	121	IVHSGDEVE	9	0.9674
FgCatL1G					
1	5	20	IDWRQYGYVTEVKNQG	16	1.8422
2	40	48	KKFRNRMLF	9	1.4887
3	57	67	TKRFGNHGCSG	11	1.9997
4	142	158	FYMYKSGIFMSQVCTTQ	17	0.5843
FgCatL1H					
1	5	19	IDWREFGYVTEVKDQ	15	1.3484
2	48	67	GDYGNRGCSG	10	3.3362

### 3.3 HTL epitope prediction

FgCatLs sequences were predicted HTL epitopes. The length of HTL epitope was 15 amino acids. The H2-I alleles, including H2-IAb and H2-IAd were used to select HTL epitopes, which were alleles of mouse. In addition, an antigenicity score of more than 0.5 was used to select HTL epitopes. The total HTL epitopes of selected FgCatL1, FgCatL1B, FgCatL1C, FgCatL1D, FgCatL1E, FgCatL1F, FgCatL1G, and FgCatL1H were 10, 10, 8, 14, 10, 9, 15, and 17 epitopes, respectively. In addition, the antigenicity score of all selected HTL epitopes ranges from 0.5095 to 1.7204 (Supplementary Table 1).

### 3.4 Selection and construction of the novel BCL and HTL epitopes

**Figure 5:**
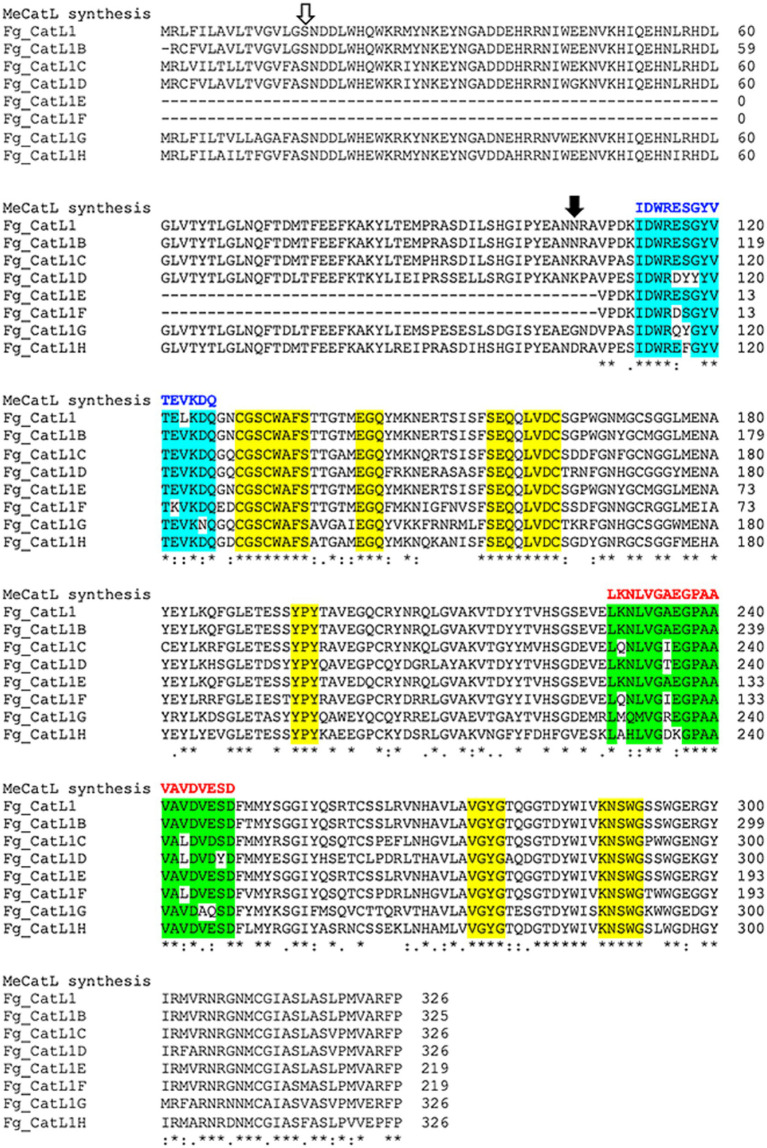
Selection and construction of the novel BCL and HTL epitope sequences. Open and solid arrows indicate the first amino acid of the pro-region and the mature protein, respectively. The yellow label indicates the conserved host’s region. Cyan and green labels indicate overlapped BCL and HTL epitope groups, respectively. Blue and red letters indicate the novel BCL and HTL sequences, respectively. Asterix (*) indicates conserved residue positions of FgCatLs, colon (:) indicates conservation between amino acid groups of similar FgCatLs properties. Period (.) indicates conservation between amino acid groups of weakly similar FgCatLs properties.

Selected BCL and HTL epitopes were marked in all FgCatLs alignments with the host’s Cathepsin L, including HsCatL, BtCatL, MmCatL, and ChCatL. The epitopes were selected from 3 criteria, including, non-conserved host’s region, overlapping amino acid sequences, and the highest percent conserved residue of epitope groups. The results of overlapping amino acid sequences showed two and one groups of BCL and HTL epitope groups, respectively. The percent conserved residue of BCL 1 (B1) and BCL 2 (B2) epitope groups showed 60 and 50 percent conserved residue, respectively. Therefore, the highest percent conserved residue of the BCL epitope group was BCL 1 (B1). The HTL epitope group was the only group that was HTL 1 (T1). The percent conserved residue of the HTL 1 (T1) epitope was 55 percent conserved residue (Supplementary Table 2). Therefore, B1 and T1 epitope groups were selected to construct the novel BCL and HTL epitopes. To construct the novel BCL and HTL epitopes, the identical amino acids of the B1 and T1 epitope groups were used to construct the novel BCL and HTL epitopes. The novel BCL and HTL sequences resulted as (IDWRESGYVTEVKDQ), and (LKNLVGAEGPAAVAVDVESD), respectively. The lengths of novel BCL and HTL were 15 aa and 20 aa, respectively (Figure 5).

### 3.5 Design of the MeCatL driven short peptide

The MeCatL driven short peptide was designed using novel BCL and HTL epitopes. The GPGPG linker was used to link the novel BCL and HTL epitopes. GPGPG, a glycine-rich protein, serves to enhance the immunological response of the host by facilitating HTL activation. The GPGPG linker molecule serves as a versatile spacer, which is capable of breaking junctional immunity. As a result, there was a restoration of immune response for each epitope (78). The final peptide sequence consisted of 40 aa containing the novel BCL epitope (15 aa), novel HTL epitope (20 aa), and GPGPG linker (5 aa). The MeCatL driven short peptide structure was shown in Figure 1.

### 3.6 Percent identity of the MeCatL driven short peptide

**Figure 6:**
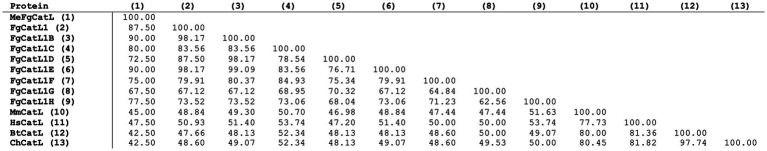
Sequence identity matrix of the MeCatL drives short peptide. The sequence identity matrix shows the sequence identity on 0–100 scale between the peptide vaccine with the mature sequences of the host’s Cathepsin L and FgCatLs.

MeCatL driven short peptide was aligned with the mature sequence of the host’s Cathepsin L and FgCatLs using the Clustal Omega v 1.2.4 server. The percent identity matrix showed 42.50%–47.50% of the host’s Cathepsin L, and 67.50%–90.00% of FgCatLs (Figure 6).

### 3.7 Prediction of physicochemical properties, antigenicity, allergenicity, toxicity, and solubility

The physicochemical properties, antigenicity, allergenicity, toxicity, and solubility of the MeCatL driven short peptide sequence were evaluated. The molecular weight of the peptide sequence was 4.13 kDa. The theoretical isoelectric point value (pI) was 4.07, representing its charge at a specific pH. The total number of negatively and positively charged residues were 8 and 3, respectively. The formula of the MeCatL driven short peptide was C_180_H_282_N_48_O_63_. In terms of peptide lifespan, the half-life was estimated to be 20 h in mammalian reticulocytes, 30 min in yeast, and >10 h in *Escherichia coli*. The instability index (II) and aliphatic index were 13.92 and 82.75, respectively, indicating that the peptide is relatively stable. The peptide is slightly hydrophilic, with a GRAVY value of −0.362. Its antigenic score was 0.8615. Overall, this vaccine sequence was found to be non-allergenic, non-toxic, and to have good solubility.

### 3.8 Prediction, refinement, and validation of tertiary structure

**Figure 7:**
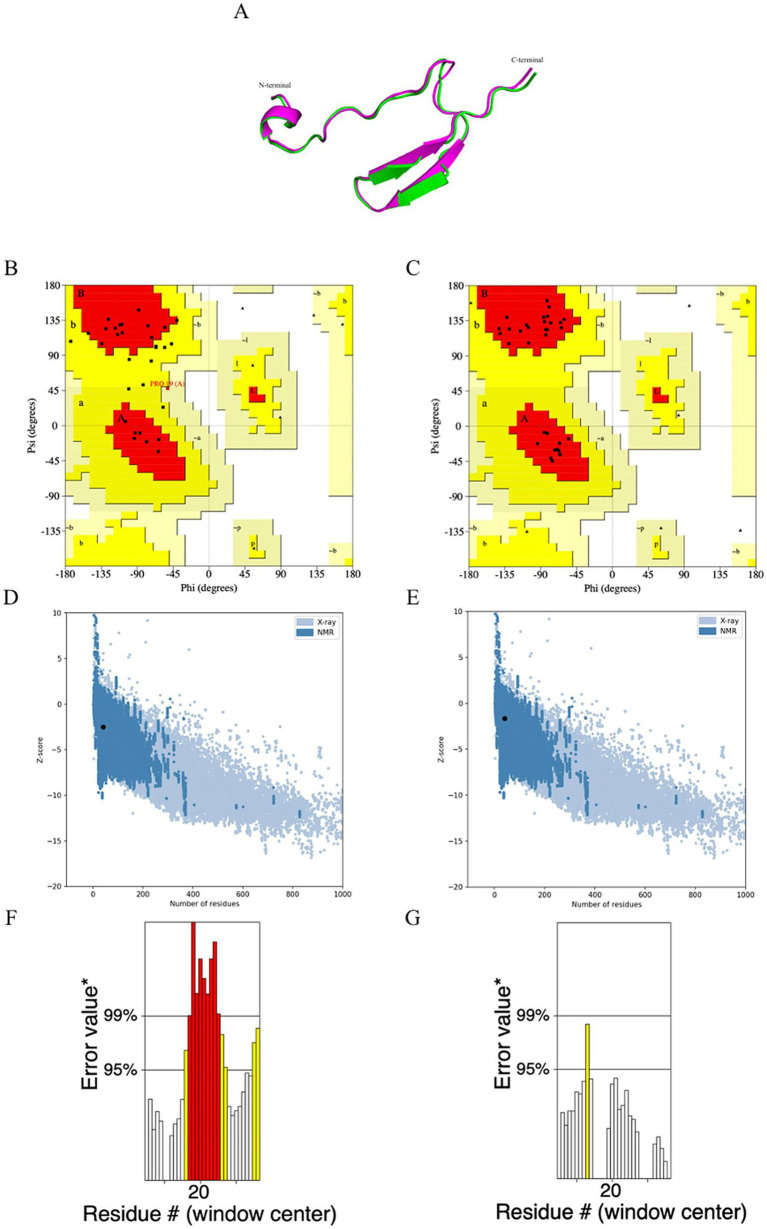
Prediction, refinement, and validation of tertiary structure. **(A)** The structural alignment of the tertiary structure obtained for the unrefined (green) and refined (magenta) MeCatL driven short peptide. **(B)** Ramachandran plot of unrefined showed 69% in the most favored region, and 31% in additional allowed region. **(C)** Ramachandran plot of the refined model showing 100% in the favored region. **(D,E)** The z-score of unrefined and refined were calculated to be −2.52, −1.66, respectively. The z-score (Black dot) indicates the overall quality of the model. Structural groups from different sources have different colors. The light blue and dark blue indicate X-ray and nuclear magnetic resonance (NMR), respectively. **(F,G)** The ERRAT score of unrefined and refined was calculated to be 51.7254 and 95.45. The ERRAT score shows overall structural quality, which is based on non-band interaction. Good structural quality produces scores of 95% or higher. Asterix (*) indicates error values. On the error axis, 95% and 99% lines indicate possible rejection at 95% and 99% confidence levels. Regions can be rejected at a 95% confidence level as yellow.

The tertiary structure of the unrefined and refined MeCatL driven short peptide models was green and magenta, respectively (Figure 7A). The Ramachandran plot of the unrefined model showed 69% in the most favored region and 31% in the additional allowed region, while the Ramachandran plot of the refined model showed 100% in the most favored region (Figures 7B,C). The refined model had a GDT-HA score of 0.8750, root mean square deviation (RMSD) score of 0.711, and MolProbity score of 2.226. The unrefined and refined models were aligned using the Pymol software v 2.5.4. The Z-scores of the unrefined and refined models were predicted as −2.52 and −1.66, respectively (Figures 7D,E). In addition, the ERRAT scores of the unrefined and refined models were 51.7254 and 95.455, respectively (Figures 7F,G). The results for the refined model suggested good structural quality.

### 3.9 Prediction of discontinuous and continuous B cell epitopes

**Figure 8:**
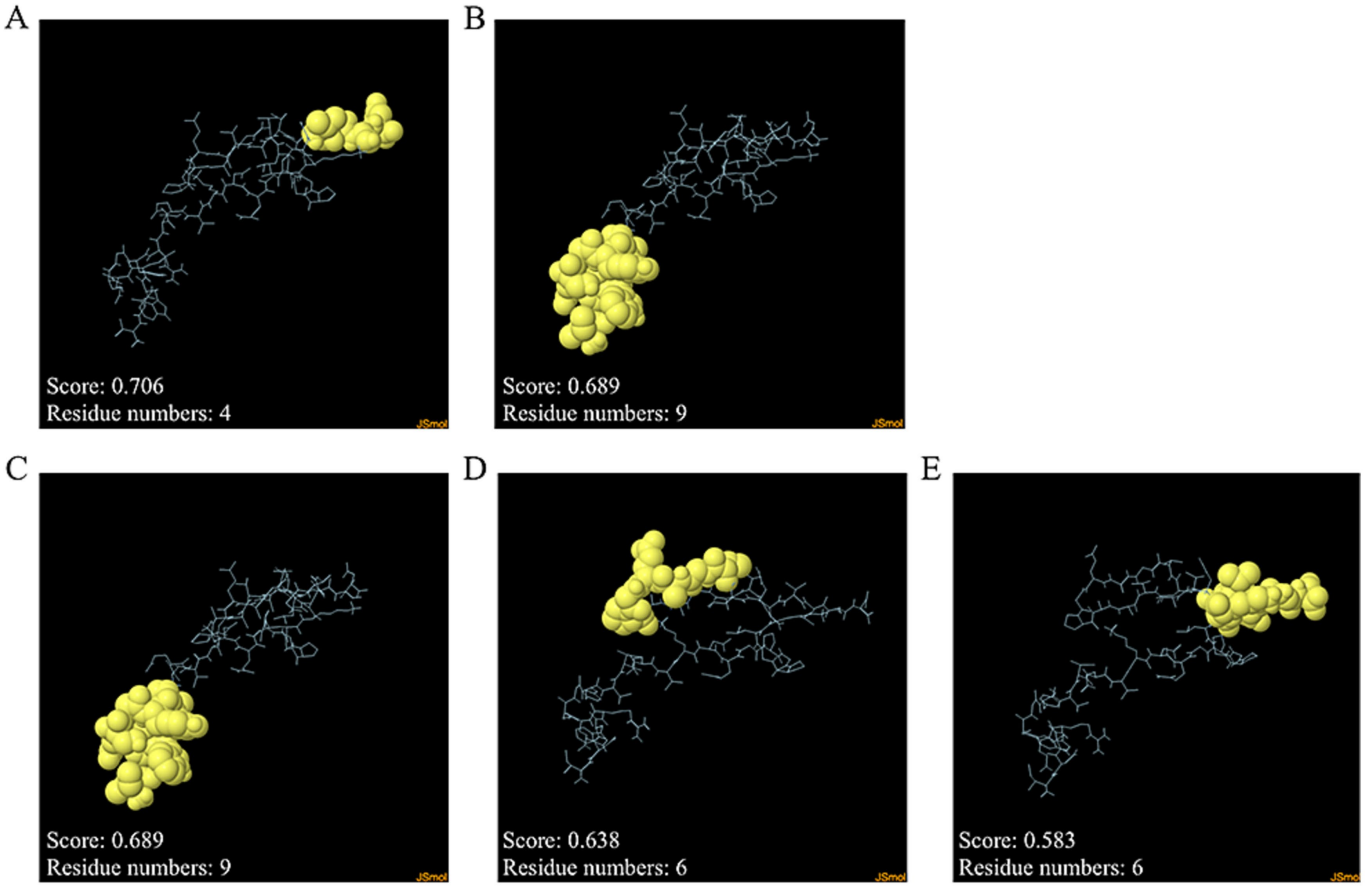
Discontinuous and continuous B cell epitopes of refined models. **(A,B)** Continuous B cell epitopes, and **(C–E)** Discontinuous B cell epitopes. The epitope residues and the rest of the sequence indicate yellow and cyan, respectively.

Discontinuous and continuous B cell epitopes were predicted by the ElliPro server. The result of the refined MeCatL driven short peptide model was two and three continuous and discontinuous B cell epitopes, respectively. The number of continuous B cell epitopes was four and nine residues with scores as 0.689 and 0.706, respectively (Figures 8A,B). In addition, the number of discontinuous B cell epitopes ranged from six to nine residues with score as 0.583 to 0.689 (Figures 8C–E).

### 3.10 Molecular docking and MD simulation of the MeCatL driven short peptide candidate with TLR-2

**Figure 9:**
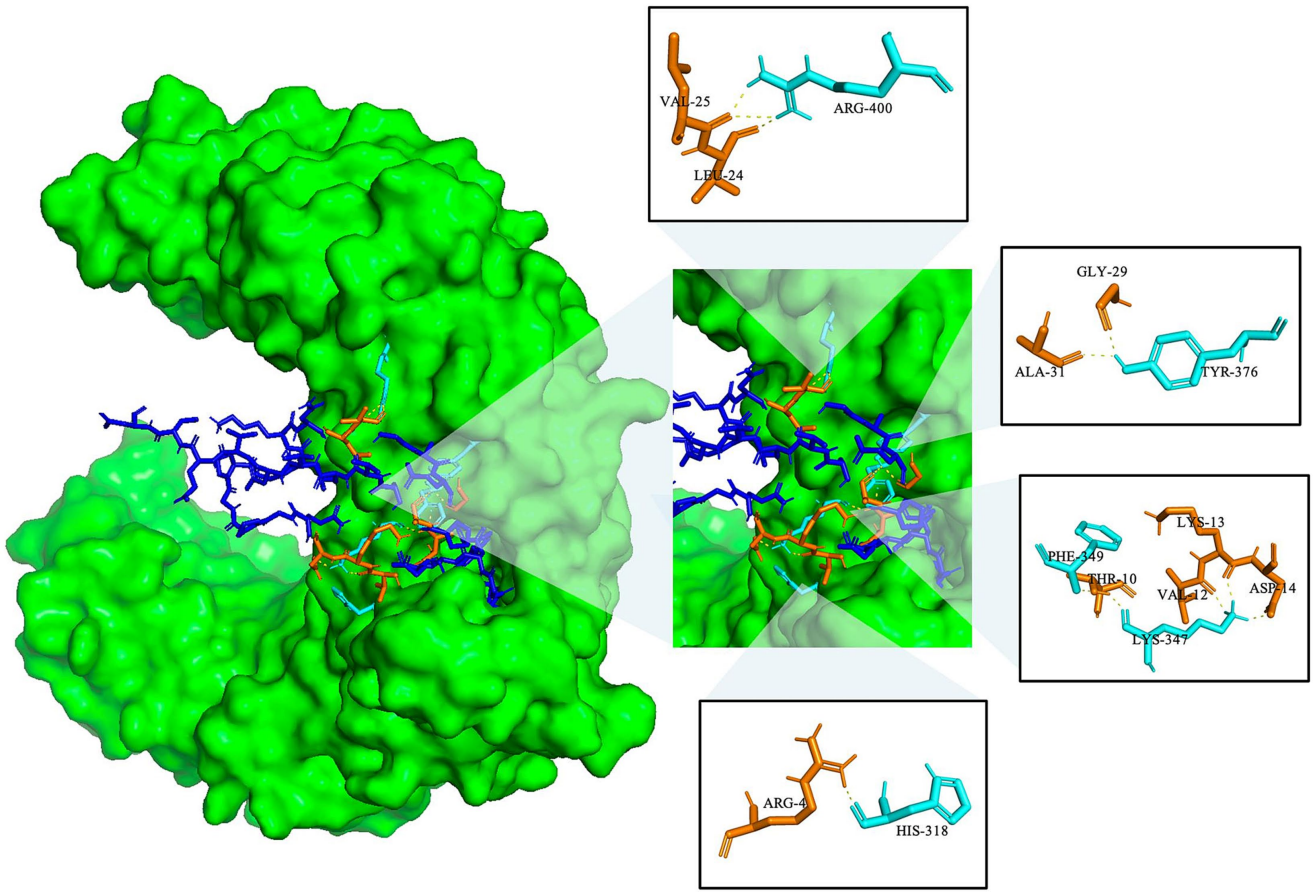
Molecular docking of the MeCatL drives a short peptide model with TLR-2. TLR-2 receptor and molecular interaction were marked as green surfaces and yellow dotted lines. Cyan and orange sticks indicate TLR-2 residue and MeCatL driven short peptide residue, respectively. The rest of the sequence indicates a blue stick.

Molecular docking was used to investigate the binding interactions between TLR-2 and the MeCatL driven short peptide model using the ClusPro2.0 server. The total cluster of the molecular docking model was 10 clusters. The model was selected from the lowest energy score, showing the highest receptor and ligand affinity. The lowest energy was −1222.4 kJ/mol. The MeCatL driven short peptide residue interaction was nine residues, including ARG4, THR10, VAL12, LYS13, ASP14, LEU24, VAL25, GLY29, and ALA31. The TLR-2 residue interaction was five residues, including HIS318, LYS347, PHE349, TYR376, and ARG400 (Figure 9).

iMODS was used to evaluate the stability and mobility of the protein docking model. The normal mode analysis (NMA) was implemented to investigate the mobility of the docked complex model, where the blue and red areas represent the lowest and highest mobility, respectively. The B-factor plot shows an average of the root mean square (RMS), and the B-factor value scores corroborate the NMA analysis. The deformability of the main chain is a measurement of the ability of a given molecule to deform each residue. The deformability index showed a lower peak distortion in the complex, reflecting a lower deformation capacity. The B-factor and deformability index results indicate the stability of the docked model complex. The eigenvalue represents the motion stiffness, which took a value of 1.840057e-05. The variance graph is inversely correlated with the eigenvalues and highlights the individual and cumulative variances in purple and green, respectively. The covariance matrix indicates the interactions between pairs of residues, where red, white, and blue colors represent correlated, uncorrelated, and anti-correlated motion, respectively. In addition, the elastic network model orders a pair of spring-bound atoms, allowing for study of the stiffness of the complex; the gray color represents a higher protein stiffness in regions. The covariance matrix and elastic network model results suggest that the docked model complex is stable (Supplement Figure 1).

### 3.11 *In-silico* immune response simulation

**Figure 10:**
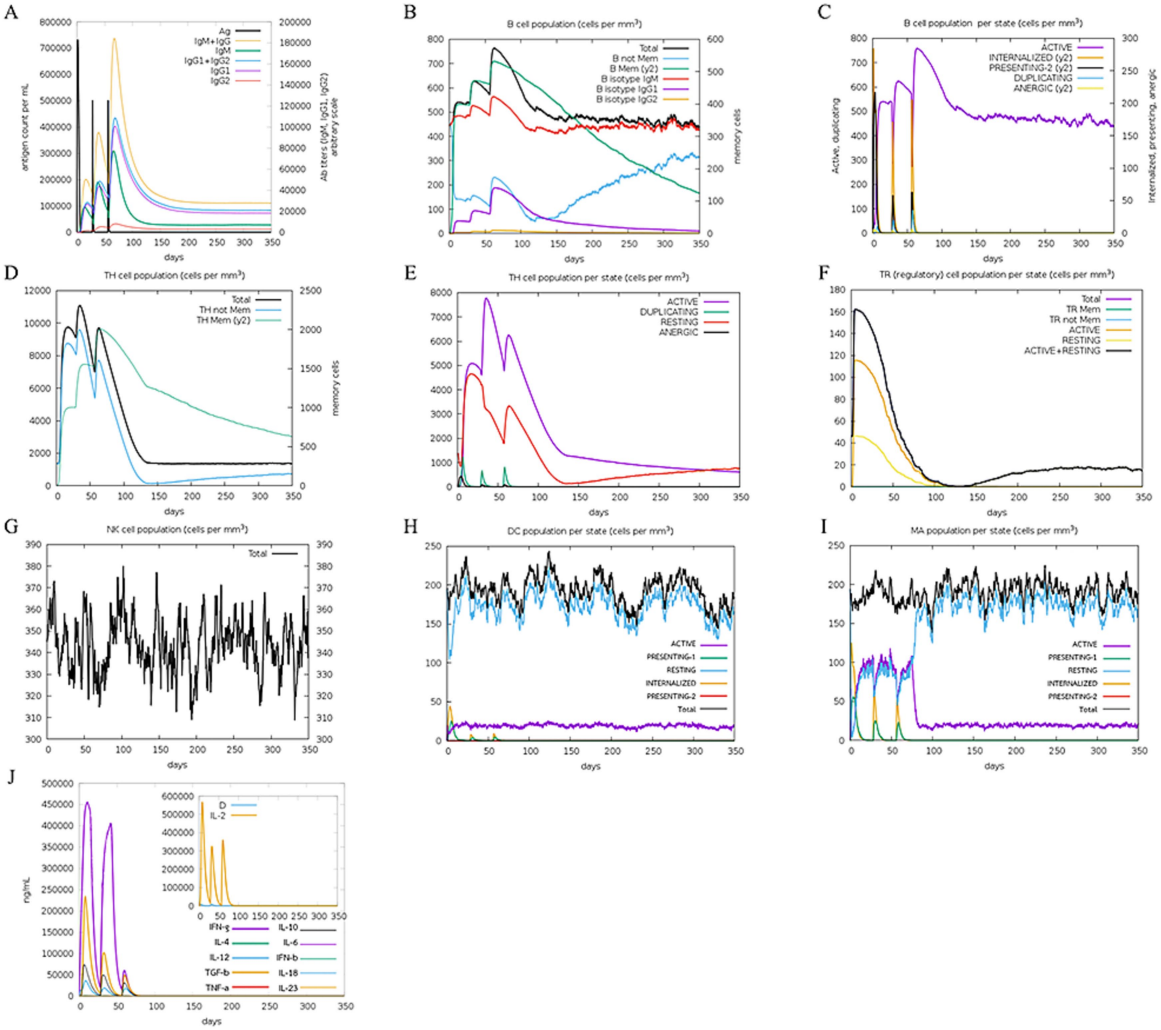
*In silico* immune response simulation. **(A)** Response of antibody and antigen, **(B)** The B-cell population, **(C)** The B-cell population per entity-state, **(D)** The T-cell population, **(E)** The T-cell population per entity-state, **(F)** The regulatory T cell population per state, **(G)** The natural killer (NK) cell population, **(H)** Macrophage (MA) population per state, **(I)** The dendritic cell (DC) population per state, **(J)** The levels of cytokines.

The immune simulation was predicted utilizing the C-immsim server. The immune response simulation results after three doses are shown in Figure 10. High levels of IgM, IgG1, and IgG2 antibodies were observed after all injections, and higher memory B cell and active B cell levels were evidenced. As such, MeCatL driven short peptide induced an extremely long-lasting immune response. Both regulatory T cells (TR) and active memory HTL provided evidence of population growth. The number of Natural Killer (NK) cells remained constant during immune simulation. A high number of active dendritic cells and macrophages were observed, suggesting effective antigen presentation. In addition, interferon gamma (IFN-*γ*), transforming growth factor beta (TGF-*β*), interleukin-10 (IL-10), and interleukin-12 (IL-12) levels were elevated.

### 3.12 IgG1 and IgG2a levels

**Figure 11:**
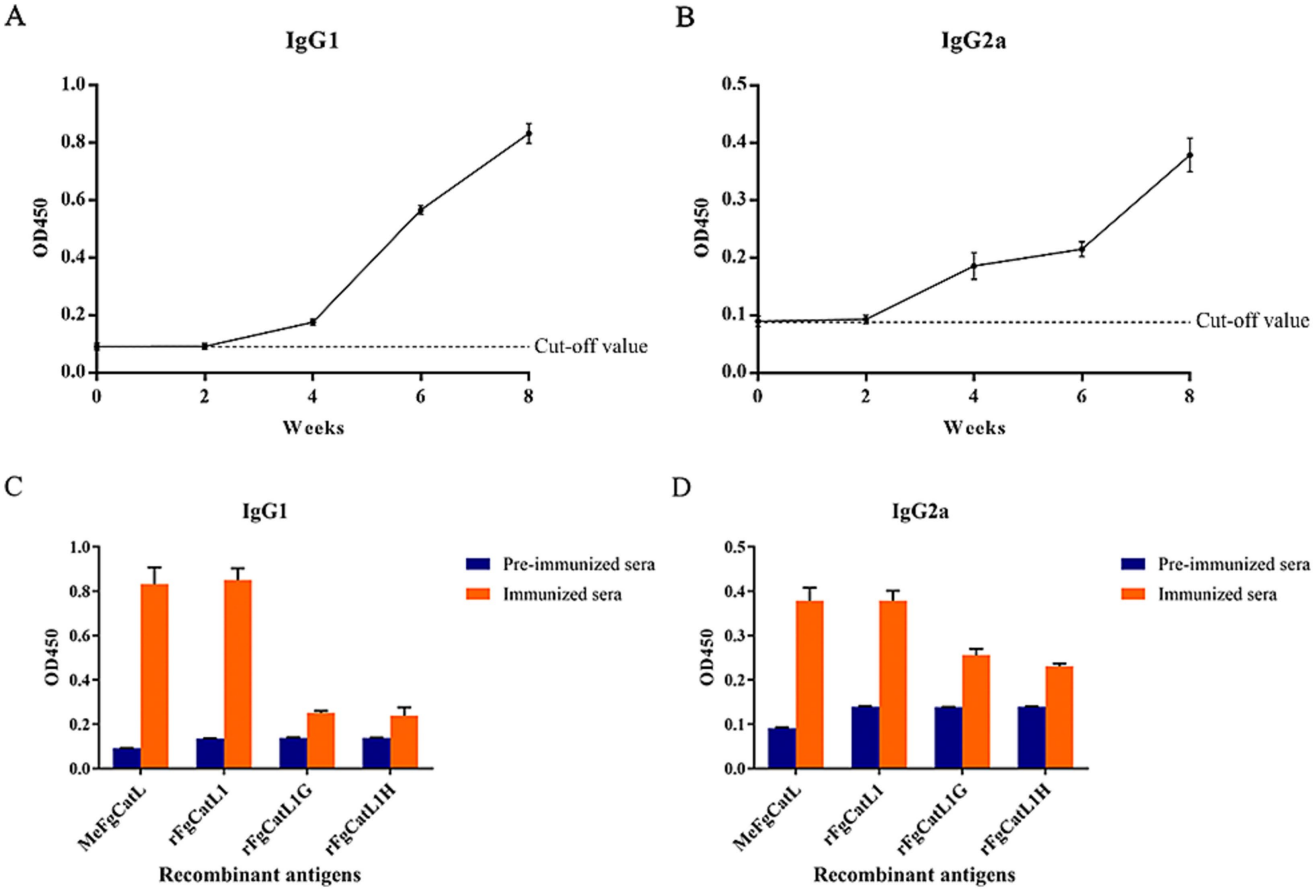
The levels of the MeCatL driven short peptide-specific antibodies. The levels of the MeFgCatL specific **(A)** IgG1 and **(B)** IgG2a. **(C)** The levels of the MeCatL driven short peptide-specific IgG1 against rFgCatL1, rFgCatL1G, rFgCatL1H, and MeCatL driven short peptide. **(D)** The levels of the MeCatL driven short peptide-specific IgG2a against rFgCatL1, rFgCatL1G, rFgCatL1H, and MeCatL driven short peptide.

Serum from pre-immunized mice and the biweekly MeCatL driven short peptide-immunized mice with Quil-A adjuvant were used to measure the IgG1 and IgG2a levels. The background in pre-immunized sera in single proteins was the OD450 values of the MeCatL driven short peptide-specific IgG1 and IgG2a levels. The MeCatL driven short peptide specific IgG1 and IgG2a levels elevated following week 2, surpassing those of the pre-immunized mice sera. rFgCatL1, rFgCatL1G, and rFgCatL1H represent proteins expressed at each stage. Week 8 immunized sera demonstrated higher levels of MeCatL driven short peptide-specific IgG1 and IgG2a against the single proteins, including rFgCatL1, rFgCatL1G, and rFgCatL1H, than pre-immunized sera (Figure 11). MeCatL driven short peptide induced both IgG1and IgG2a responses, indicating IgG1 levels were predominant.

### 3.13 Immunolocalization of *Fasciola gigantica* tissue

**Figure 12:**
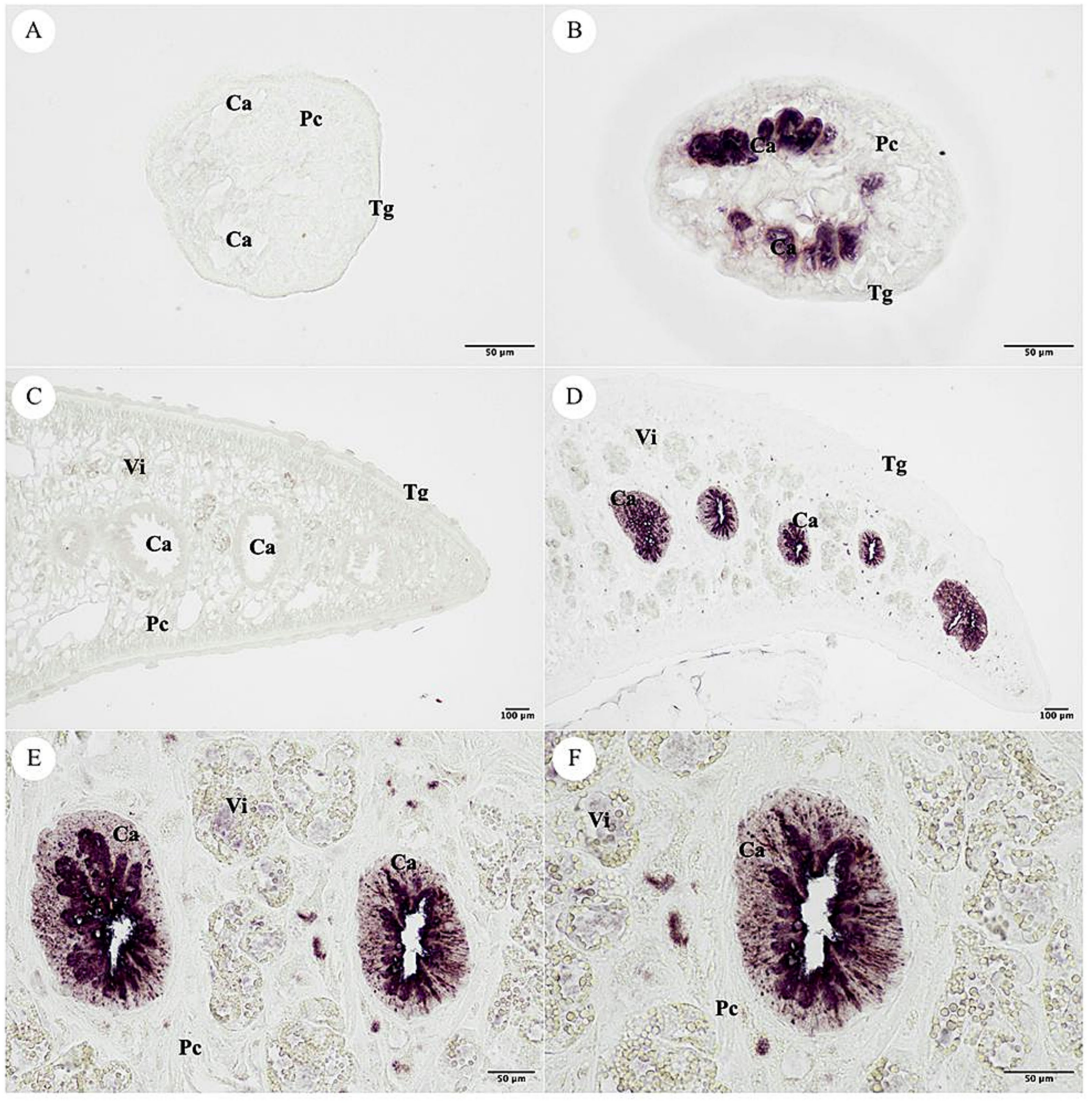
Localization of the MeCatL drives short peptide immunized mice serum. **(A)** The pre-immunized mice serum was used for negative control in the NEJ stage of *Fasciola gigantica* tissue. **(B)** The MeCatL driven short peptide immunized mice serum indicated the cecal epithelium cell (Ca) in the NEJ stage of *F. gigantica* tissue. The tegumental cell (Tg), parenchyma (Pc) and vitelline gland (Vi) were unstaining. **(C)** The pre-immunized mice serum was used for negative control in the adult stage of *F. gigantica* tissue. **(D–F)** The MeCatL driven short peptide immunized mice serum indicated the cecal epithelium cell (Ca) in the adult stage of *F. gigantica* tissue. The tegumental cell (Tg), parenchyma (Pc) and vitelline gland (Vi) were unstaining.

The NEJ and adult *F. gigantica* tissue sections were used for immunolocalization. The negative control used pre-immunized mice sera that was not stained in the *F. gigantica* section. The positive signal was specifically detected in the cecal epithelium cell (Ca) of the NEJ, and adult *F. gigantica* tissues. The tegumental cell (Tg), parenchyma (Pc) and vitelline gland (Vi) were not stained (Figure 12).

## 4 Discussion

*Fasciola* spp. infection plays an essential role in public health. Many years ago, the development of vaccines against fasciolosis was initiated, facing various challenges. Many types of vaccine have been tested against *Fasciola* spp., such as single recombinant protein vaccines (22), combined recombinant protein vaccines (79), and phage display-based vaccines (80). Prior research has indicated that numerous vaccines exhibit high levels of efficacy in protecting against *Fasciola* spp. infection (11, 14, 16, 18, 23, 24). However, many vaccines are manufactured using bacteria or yeast, which might result in bacterial or yeast contamination. Furthermore, the associated production method is intricate, and the production volume is limited (42). Peptide vaccines (or epitope vaccines) are a new technology that allows for the selection of specific immunogenic epitopes using immunoinformatic tools. Therefore, peptide vaccine design can be considered an essential tool for the development of fasciolosis vaccines.

In the present study, the MeCatL driven short peptide was designed using immunoinformatic tools. In particular, online servers were used to predict the BCL and HTL epitopes, where the number of selected BCL and HTL epitopes for FgCatLs ranged from 2 to 4 epitopes and 8 to 17 epitopes, respectively. In addition, the selected BCL and HTL epitopes had predicted antigenic scores from 0.549 to 3.3362 and 0.5095 to 1.7204, respectively. The length of the selected BCL epitopes ranged from 6 to 16 aa, while the length of the selected HTL epitopes was 15 aa; for comparison, in previous studies, the lengths of BCL and HTL epitopes ranged from 5 to 22 and 15 to 24 aa, respectively (81, 82). All epitopes were marked in all FgCatLs alignments with the host’s Cathepsin L, including HsCatL, BtCatL, MmCatL, and ChCatL. The criteria for the selection of epitopes to construct the novel vaccine included a non-conserved host region, overlapping sequence, and the highest percent conserved residue of overlapped epitopes. Conserved regions are similar protein sequences in the cross-species context. For this study, we selected only non-conserved host regions, as the selected epitope must not contain similar host sequences. However, the selected BCL and HTL epitopes similarly contained sequences of all FgCatLs. In addition, overlapping sequences and the highest percent conserved residue of overlapped epitopes helped to construct the novel epitopes, which were used to select amino acids for construction of the novel epitopes. Therefore, the novel BCL and HTL epitopes were combined isotypes of FgCatLs, which were used to design the peptide vaccine. The novel BCL and HTL sequences were (IDWRESGYVTEVKDQ) and (LKNLVGAEGPAAVAVDVESD), respectively. The matching of the novel epitope sequences with other isotype proteins results in a vaccine’s possible blocking activity against other isotype proteins and ability to prevent infection with other parasite species, also called cross-protection. Cross-protection against related non-vaccine microorganism types is a vaccine concept that has recently attracted attention in the disease prevention field (83).

The MeCatL driven short peptide sequence was linked using the GPGPG linker. Linkers are short amino acid sequences, which are used to separate epitopes in the peptide sequence. Linkers are crucial in preventing neo-epitope generation, which is an important concern when designing epitope vaccines. In addition, linkers are generally selected for sequences that cannot stimulate the immune system (84, 85). In this study, the GPGPG linker was used to differentiate epitopes, separating B cell and HTL epitopes. The GPGPG linker (glycine-rich) is able to induce HTL responses and protein folding, as well as increase solubility and flexibility, and make protein structure more stable (86, 87).

Protein identity searches for homologous sequences were conducted in a database (88, 89). The protein identity matrix obtained through these tests revealed that the peptide vaccine exhibited a range of 42.50%–47.50% similarity to the host Cathepsin L and 67.50–90.00% similarity to FgCatLs. Therefore, the peptide vaccine had low similarity to the host Cathepsin L proteins. On the other hand, the peptide sequence had high similarity to FgCatLs proteins. In a previous study, an epitope peptide vaccine against *Bunyamwera orthobunyavirus* presented similarities to the whole sequences of M-polyprotein (90). This similarity will likely result in immunological cross-reactivity, where the immune system’s response to the harmful antigens of the pathogen may damage comparable proteins in the host, ultimately leading to autoimmune disease (81–93).

Various physicochemical properties of the peptide sequence were analyzed. According to the results, the molecular weight and pI value were 4.13 kDa and 4.07, respectively. The half-life was estimated to be 20 h in mammalian reticulocytes, 30 min in yeast, and >10 h in *Escherichia coli*. The instability index (II) was 13.92. The protein was stable (II value < 40) (94). The aliphatic index and GRAVY value were 82.75 and −0.362, respectively. Antigenicity denotes the ability of a protein to induce an immune response in a competent host, which depends on exogenous factors such as the host’s immunoglobulins, self-tolerance, cytokine production, and various cellular and regulatory mechanisms (95). The antigenic score of the constructed peptide vaccine was 0.8615. In addition, the peptide vaccine was non-allergenicity, non-toxicity, and suitably soluble.

The AlphaFold2 server was used to predict the tertiary structure of the MeCatL driven short peptide. The highest pLDDT score was selected for refinement using the GalaxyRefine server as the final step to improve the quality of the predicted protein models (96). The Ramachandran plot was used to assess the accuracy of the predicted protein structure, allowing for the prediction of stereochemical structural properties (97). The Ramachandran plots for the refined model indicated a more improved model than the unrefined model, and the Z-score of refined models was calculated to be −1.66. Therefore, the MeCatL driven short peptide model fell within the typical range of NMR-solved protein structures (69, 98). The ERRAT score indicates the overall quality of a structure based on non-band interactions. Good structural quality produces scores of 95% or higher. The refined model’s ERRAT score was calculated to be 95.45. All the obtained results indicated that the MeCatL driven short peptide model has good structural qualities.

During the initial stages of *F. gigantica* infection, B cells play a role in the production of neutralizing antibodies that target liver flukes. Consequently, the parasite is unable to survive within the host’s body (34). Discontinuous and continuous B cell epitopes were used to predict epitopes using the EliPro server. The ElliPro server predicts discontinuous and continuous B cell epitopes based on the 3D structure of the antigen protein (70). The peptide model was used to assess the presence of discontinuous and continuous B cell epitopes, which predicts the binding to proteins responsible for the host immune response. Most B cell epitopes (approximately 90%) are discontinuous, and only a minority are continuous (99–101). The designed MeCatL driven short peptide was constructed from continuous B cell epitopes; however, the designed vaccine can also predict discontinuous epitopes (70, 95). In this study, two and three epitopes were continuous and discontinuous B cell epitopes, respectively. These epitopes are likely able to stimulate B cell binding and recognition. In addition, TLR-2 plays a key role in the immune response and promotes the immune response. The TLR-2 receptor has been used in previous studies investigating the interactions between fasciolosis vaccines (102–104). The MeCatL driven short peptide model was docked with TLR-2 using the ClusPro2.0 server, which showed that the vaccine was capable of interacting with immunological receptors (105). The lowest energy score of the docked model indicates the highest binding affinity (106). The lowest energy score was −1222.4 kJ/mol; hence, the designed vaccine possesses the capacity to stimulate both innate and adaptive immunity. The Th2 immune response is activated through the TLR-2 receptor on the surface of B cells and CD4 + T cells (107). The TLR-2 receptor induces the ERK1/2 signaling pathway, resulting in promotion of the transcription factor c-Fos, thus suppressing IL-12 production and promoting IL-10 secretion to induce Th2 type-immune responses (108). Th2 cells secrete cytokines that trigger eosinophils, stimulate immunoglobulin production, and the secretion of inflammatory mediators (35). Consequently, the MeCatL driven short peptide was effective in inducing host immunity through the TLR-2 receptor. MD simulation of the docked model complex was investigated using iMODS server, and the results indicated good stability.

The constructed peptide vaccine is likely able to activate the immune system of the host and provide protection against *F. gigantica* infection. Prior research has involved the evaluation of predicted peptide vaccines against parasites through *in vivo* testing, demonstrating their ability to stimulate an immune response and provide protection against parasite infection (51, 55–59). In this study, the C-ImmSim server was used to evaluate the efficacy of predicted peptide vaccines in generating an immune response. Through *in-silico* testing, the constructed peptide vaccine was found to be capable of inducing both B cell-and T cell-mediated responses, and the IgM and IgG antibody levels increased after the primary dose. Memory B cells were also observed, indicating a long-lasting immune response (109). The population of active HTLs increased after the first dose, indicating effective stimulation of cell-mediated immunity. In addition, the populations of active dendritic cells and macrophages were highly elevated. These results suggest that the designed peptide vaccine can mimic the natural immunity induced by antigens (110). Furthermore, cytokines were triggered by the MeFgCatL vaccine. There was an increase in TGF-*β*, IL-10, IL-12, and IFN-*γ*. M2 macrophages generated TGF-β and IL-10, which enhanced the proliferation of Th2 and T regulator (Treg) cells (35, 111). IL-12 has the ability to stimulate the differentiation of Th1 cells. However, Th1 cells differentiation is inhibited by a parasite survival mechanism (35). In addition, Th1 cell released IFN-γ, which can aid in the death of parasites (111, 112). Increasing IFN-γ and IL-12 will most likely aid in the mechanism for clearing parasites. Previous *in-silico* investigations using immunoinformatic approaches have demonstrated the ability of *Fasciola* spp. multi-epitope vaccines to stimulate T and B cell immune responses (104).

Adjuvants have a crucial role in imitating the host immune response. *Quillaja saponaria* bark is used to extract Quil-A adjuvant, an aqueous saponin. Aqueous saponin can generate strong immune responses to antigens that are both T-dependent and T-independent (112). In both human and livestock vaccination trials, Quil-A adjuvant is utilized to induce a mixed Th1/Th2 immune response (113–115). The IgG1 was produced by the Th2 immune response. On the other hand, the IgG2a level was produced by the Th1 immune response (116). The IgG1 and IgG2a levels increased at week two sera compared to pre-immunized mice sera. MeCatL driven short peptide with Quil-A adjuvant produced a mixed Th1/Th2 immune response, suggesting a predominately Th2 immune response. In previous studies, an increase in parasite-specific serum antibody activity has been associated with the Th2 immune response (117–119). Therefore, the IgG1 level was highly increased compared with the IgG2a level. The three proteins, rFgCatL1, rFgCatL1G, and rFgCatL1H represent the proteins expressed at each stage. In the adult stage, FgCatL1 was shown to be mainly expressed (31). FgCatL1G was majorly expressed in NEJ and juvenile stages (24, 120). FgCatL1H were found mainly in the juvenile stage (23). The IgG1 and IgG2a levels were detected with rFgCatL1, rFgCatL1G, and rFgCatL1H. The immunological responses in mice were similar in *in silico* immune simulation. The MeCatL drives short peptide to elicit cellular and humoral immune responses. In addition, the reactivity of the MeCatL driven short peptide with rFgCatL1 strongly increased compared with rFgCatL1G, and rFgCatL1H that MeCatL driven short peptide was similar the overlapping BCL and HTL epitope sequences of rFgCatL1 more than rFgCatL1G and rFgCatL1H. The MeCatL driven short peptide immunized sera was used to localize the *F. gigantica* tissue. The result showed it specifically detected in the caecal epithelium of NEJ and adult *F. gigantica* tissues. The major organ where cathepsins are expressed is the caecal epithelium (31). Therefore, the MeCatL driven short peptide able to affect the parasite’s digestion capacity. These results were consistent with previous recombinant vaccine studies, including rFgCatL1 (31), rFgCatL1G (24), and rFgCatL1H (23).

Although the results of this study demonstrate promising, several limitations should be considered. In particular, the long-term stability of peptide-based vaccines was not evaluated. Peptide vaccines are known to degrade rapidly if not stored properly, which could impact their effectiveness. Additionally, the long-term safety of the vaccine remains a concern. Although no immediate side effects were observed, experimental animals exhibited potential indicators of risk, such as hypersensitivity and autoimmunity. Moreover, the study was conducted under controlled laboratory conditions using an animal model, which may not fully reflect the complexity of natural *F. gigantica* infection. Nevertheless, ongoing studies are assessing the protective efficacy of the MeCatL-driven short peptide against *F. gigantica* infection.

## 5 Conclusion

In this study, the MeCatL driven short peptide indicated good quality structure. In the mice experiment, the levels of IgG1 and IgG2a antibodies were increased after week 2, suggesting a mixed Th1/Th2 immune response, with a predominance of Th2. Sera from mice immunized with the MeCatL-driven short peptide successfully recognized rFgCatL1, rFgCatL1G, and rFgCatL1H proteins. In addition, the immunized mice sera specifically bound to the cecal epithelium of newly excysted juveniles (NEJ) and adult *F. gigantica*. These findings indicate that the MeCatL-driven short peptide is capable of eliciting a specific immune response in the host. However, the protective efficacy of this peptide against fasciolosis remains to be investigated in future studies.

## Data Availability

The datasets presented in this study can be found in online repositories. The names of the repository/repositories and accession number(s) can be found in the article/Supplementary material.

## Footnotes

^1^ https://www.ncbi.nlm.nih.gov/

^2^ https://web.expasy.org/protparam/

^3^ https://www.ddg-pharmfac.net/AllerTOP/

^4^ http://scratch.proteomics.ics.uci.edu/

^5^ https://pepcalc.com

^6^ http://tools.iedb.org/main/bcell/

^7^ http://tools.iedb.org/mhcii/

^8^ https://www.ebi.ac.uk/Tools/msa/clustalo/

^9^ https://github.com/sokrypton/ColabFold

^10^ http://galaxy.seoklab.org/cgi-bin/submit.cgi?type=REFINE

^11^ https://saves.mbi.ucla.edu

^12^ https://prosa.services.came.sbg.ac.at/prosa.php

^13^ http://tools.iedb.org/ellipro/

^14^ https://cluspro.bu.edu/login.php

^15^ https://imods.iqfr.csic.es

^16^ https://kraken.iac.rm.cnr.it/C-IMMSIM
