# Supplementary material for: Development of multi-epitope Cathepsin L driven short peptide vaccine against Fasciola gigantica
Source: Front Vet Sci. 2025 May 22;12:1547937. doi: 10.3389/fvets.2025.1547937 (PMC12139528; doi:10.3389/fvets.2025.1547937)
Supplement: Supplementary file 1 [file Supplementary_Image_1.pdf]

Supplementary Material

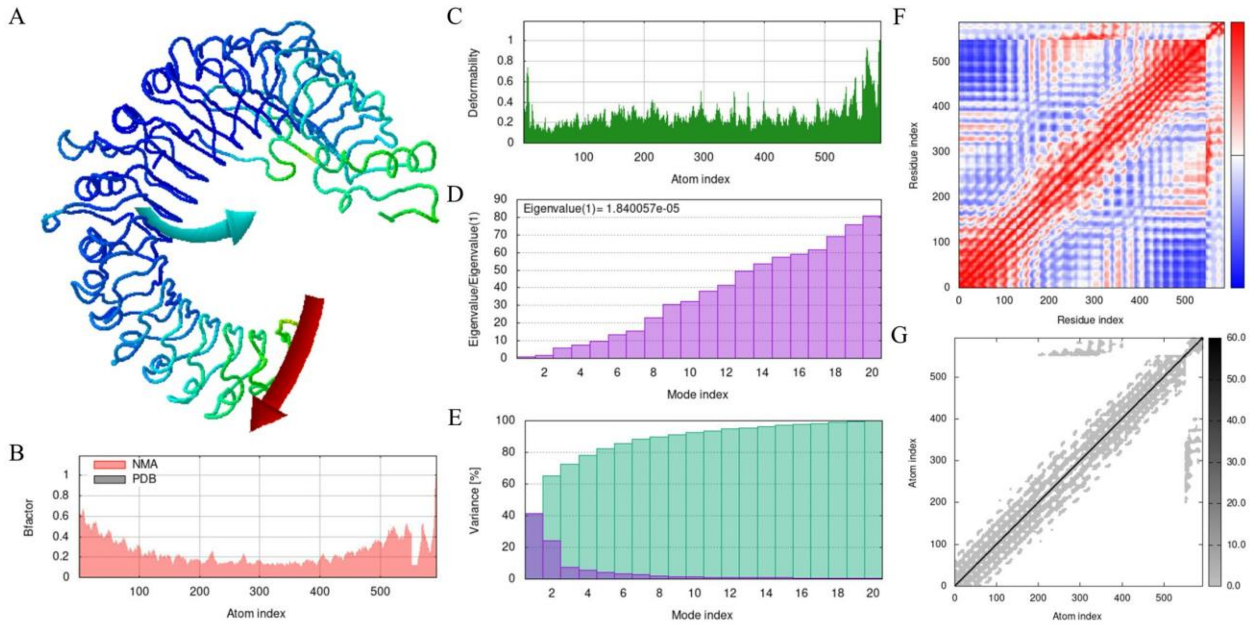

Supplementary Figure 1. MD simulation analysis. (A) The MeCatL driven short peptide model and TLR-2 docking complex. The blue and red arrows indicate the mobility, where the longer arrows indicate greater mobility. (B) The B-factor is a regional mobility score ranging from 0 (lowest) to 1 (highest). (C) The deformability index of the individual residues in the complex with lower distortion. (D) The eigenvalue is the motion stiffness as 1.840057e-05 (E) The variance graph is inversely correlated with the eigenvalues. Green and purple colors indicate cumulative and individual variances. (F) The covariance matrix is the interaction between pairs of residues. Red, white, and blue colors indicate correlated, uncorrelated, and anti-correlated motions, respectively. (G) The elastic network model is pair of atoms connection. The grey color indicates a higher protein stiffness in regions.
