# Supplementary material for: Development of multi-epitope Cathepsin L driven short peptide vaccine against Fasciola gigantica
Source: Front Vet Sci. 2025 May 22;12:1547937. doi: 10.3389/fvets.2025.1547937 (PMC12139528; doi:10.3389/fvets.2025.1547937)
Supplement: Supplementary file 2 [file Supplementary_Table_1.DOCX]

Supplementary Material

**Supplementary Table 1.** Selected HTL epitopes for mature *F. gigantica* sequences.

| **No.** | **Allele** | **Start** | **Stop** | **Peptide Sequences** | **Antigenicity** |
| --- | --- | --- | --- | --- | --- |
| **FgCatL1** | | | | | |
| 1 | H2-IAb | 125 | 139 | LVGAEGPAAVAVDVE | 1.0107 |
| 2 | H2-IAb | 124 | 138 | NLVGAEGPAAVAVDV | 0.7370 |
| 3 | H2-IAb | 123 | 137 | KNLVGAEGPAAVAVD | 0.7382 |
| 4 | H2-IAb | 126 | 140 | VGAEGPAAVAVDVES | 1.2236 |
| 5 | H2-IAb | 127 | 141 | GAEGPAAVAVDVESD | 1.4477 |
| 6 | H2-IAd | 128 | 142 | AEGPAAVAVDVESDF | 1.0614 |
| 7 | H2-IAd | 126 | 140 | VGAEGPAAVAVDVES | 1.2236 |
| 8 | H2-IAd | 127 | 141 | GAEGPAAVAVDVESD | 1.4477 |
| 9 | H2-IAd | 125 | 139 | LVGAEGPAAVAVDVE | 1.0107 |
| 10 | H2-IAd | 129 | 143 | EGPAAVAVDVESDFM | 1.0365 |
| **FgCatL1B** | | | | | |
| 1 | H2-IAb | 125 | 139 | LVGAEGPAAVAVDVE | 1.0107 |
| 2 | H2-IAb | 124 | 138 | NLVGAEGPAAVAVDV | 0.7370 |
| 3 | H2-IAb | 123 | 137 | KNLVGAEGPAAVAVD | 0.7382 |
| 4 | H2-IAb | 126 | 140 | VGAEGPAAVAVDVES | 0.7382 |
| 5 | H2-IAb | 127 | 141 | GAEGPAAVAVDVESD | 1.4477 |
| 6 | H2-IAd | 129 | 143 | EGPAAVAVDVESDFT | 0.9727 |
| 7 | H2-IAd | 128 | 142 | AEGPAAVAVDVESDF | 1.0614 |
| 8 | H2-IAd | 126 | 140 | VGAEGPAAVAVDVES | 1.2236 |
| 9 | H2-IAd | 127 | 141 | GAEGPAAVAVDVESD | 1.4477 |
| 10 | H2-IAd | 125 | 139 | LVGAEGPAAVAVDVE | 1.0107 |
| **FgCatL1C** | | | | | |
| 1 | H2-IAb | 125 | 139 | LVGIEGPAAVALDVD | 0.9402 |
| 2 | H2-IAb | 124 | 138 | NLVGIEGPAAVALDV | 0.5390 |
| 3 | H2-IAb | 126 | 140 | VGIEGPAAVALDVDS | 1.2233 |
| 4 | H2-IAb | 123 | 137 | QNLVGIEGPAAVALD | 0.5177 |
| 5 | H2-IAb | 127 | 141 | GIEGPAAVALDVDSD | 1.3954 |
| 6 | H2-IAd | 126 | 140 | VGIEGPAAVALDVDS | 1.2233 |
| 7 | H2-IAd | 125 | 139 | LVGIEGPAAVALDVD | 0.9402 |
| 8 | H2-IAb | 23 | 38 | SCWAFSTTGAMEGQY | 0.6280 |
| **FgCatL1D** | | | | | |
| 1 | H2-IAb | 125 | 139 | LVGTEGPAAVALDVD | 0.9649 |
| 2 | H2-IAb | 124 | 138 | NLVGTEGPAAVALDV | 0.5992 |
| 3 | H2-IAb | 126 | 140 | VGTEGPAAVALDVDY | 1.0793 |
| 4 | H2-IAb | 123 | 137 | KNLVGTEGPAAVALD | 0.7002 |
| 5 | H2-IAd | 38 | 52 | FRKNERASASFSEQQ | 0.8461 |
| 6 | H2-IAb | 7 | 21 | WRDYYYVTEVKDQGQ | 1.4929 |
| 7 | H2-IAd | 37 | 51 | QFRKNERASASFSEQ | 1.0030 |
| 8 | H2-IAd | 39 | 53 | RKNERASASFSEQQL | 0.8729 |
| 9 | H2-IAb | 26 | 40 | WAFSTTGAMEGQFRK | 0.5095 |
| 10 | H2-IAb | 127 | 141 | GTEGPAAVALDVDYD | 1.2067 |
| 11 | H2-IAb | 6 | 20 | DWRDYYYVTEVKDQG | 1.3290 |
| 12 | H2-IAd | 126 | 140 | VGTEGPAAVALDVDY | 1.0793 |
| 13 | H2-IAd | 125 | 139 | LVGTEGPAAVALDVD | 0.9649 |
| 14 | H2-IAb | 25 | 39 | CWAFSTTGAMEGQFR | 0.6103 |
| **FgCatL1E** | | | | | |
| 1 | H2-IAb | 125 | 139 | LVGAEGPAAVAVDVE | 1.0107 |
| 2 | H2-IAb | 124 | 138 | NLVGAEGPAAVAVDV | 0.7370 |
| 3 | H2-IAb | 123 | 137 | KNLVGAEGPAAVAVD | 0.7382 |
| 4 | H2-IAb | 126 | 140 | VGAEGPAAVAVDVES | 1.2236 |
| 5 | H2-IAb | 127 | 141 | GAEGPAAVAVDVESD | 1.4477 |
| 6 | H2-IAd | 128 | 142 | AEGPAAVAVDVESDF | 1.0614 |
| 7 | H2-IAd | 126 | 140 | VGAEGPAAVAVDVES | 1.2236 |
| 8 | H2-IAd | 127 | 141 | GAEGPAAVAVDVESD | 1.4477 |
| 9 | H2-IAd | 125 | 139 | LVGAEGPAAVAVDVE | 1.0107 |
| 10 | H2-IAd | 129 | 143 | EGPAAVAVDVESDFM | 1.0365 |
| **FgCatL1F** | | | | | |
| 1 | H2-IAb | 125 | 139 | LVGIEGPAAVALDVE | 0.8709 |
| 2 | H2-IAb | 124 | 138 | NLVGIEGPAAVALDV | 0.5390 |
| 3 | H2-IAb | 126 | 140 | VGIEGPAAVALDVES | 1.1402 |
| 4 | H2-IAb | 123 | 137 | QNLVGIEGPAAVALD | 0.5177 |
| 5 | H2-IAb | 127 | 141 | GIEGPAAVALDVESD | 1.2764 |
| 6 | H2-IAb | 145 | 159 | YRSGIYQSQTCSPDR | 0.5357 |
| 7 | H2-IAd | 126 | 140 | VGIEGPAAVALDVES | 1.1402 |
| 8 | H2-IAd | 125 | 139 | LVGIEGPAAVALDVE | 0.8709 |
| 9 | H2-IAd | 129 | 143 | EGPAAVALDVESDFV | 0.8644 |
| **FgCatL1G** | | | | | |
| 1 | H2-IAd | 128 | 142 | REGPAAVAVDAQSDF | 0.7586 |
| 2 | H2-IAd | 156 | 170 | TTQRVTHAVLAVGYG | 0.1434 |
| 4 | H2-IAd | 129 | 143 | EGPAAVAVDAQSDFY | 0.9045 |
| 5 | H2-IAd | 127 | 141 | GREGPAAVAVDAQSD | 1.2801 |
| 6 | H2-IAb | 125 | 139 | MVGREGPAAVAVDAQ | 0.7856 |
| 7 | H2-IAd | 130 | 144 | GPAAVAVDAQSDFYM | 0.8252 |
| 8 | H2-IAb | 124 | 138 | QMVGREGPAAVAVDA | 0.7492 |
| 9 | H2-IAd | 126 | 140 | VGREGPAAVAVDAQS | 0.8755 |
| 10 | H2-IAb | 126 | 114 | VGREGPAAVAVDAQS | 0.8755 |
| 12 | H2-IAb | 123 | 137 | MQMVGREGPAAVAVD | 0.6520 |
| 13 | H2-IAd | 158 | 172 | QRVTHAVLAVGYGTE | 0.5105 |
| 14 | H2-IAd | 125 | 139 | MVGREGPAAVAVDAQ | 0.7856 |
| 15 | H2-IAb | 7 | 21 | WRQYGYVTEVKNQGQ | 1.7204 |
| **FgCatL1H** | | | | | |
| 1 | H2-IAd | 75 | 89 | EYLYEVGLETESSYP | 0.5142 |
| 2 | H2-IAb | 125 | 139 | LVGDKGPAAVAVDVE | 0.9112 |
| 3 | H2-IAb | 124 | 138 | HLVGDKGPAAVAVDV | 0.6198 |
| 4 | H2-IAb | 126 | 140 | VGDKGPAAVAVDVES | 0.9716 |
| 5 | H2-IAb | 123 | 137 | AHLVGDKGPAAVAVD | 0.6655 |
| 6 | H2-IAd | 36 | 50 | GQYMKNQKANISFSE | 0.7511 |
| 7 | H2-IAd | 35 | 49 | EGQYMKNQKANISFS | 0.8294 |
| 8 | H2-IAb | 127 | 141 | GDKGPAAVAVDVESD | 1.3781 |
| 9 | H2-IAd | 128 | 142 | DKGPAAVAVDVESDF | 0.8588 |
| 10 | H2-IAb | 24 | 38 | SCWAFSATGAMEGQY | 0.6528 |
| 11 | H2-IAd | 34 | 48 | MEGQYMKNQKANISF | 0.9484 |
| 12 | H2-IAd | 127 | 141 | GDKGPAAVAVDVESD | 1.3781 |
| 13 | H2-IAd | 37 | 51 | QYMKNQKANISFSEQ | 0.6040 |
| 14 | H2-IAb | 26 | 40 | WAFSATGAMEGQYMK | 0.6530 |
| 15 | H2-IAd | 119 | 133 | ESKLAHLVGDKGPAA | 0.5264 |
| 16 | H2-IAb | 25 | 39 | CWAFSATGAMEGQYM | 0.5578 |
| 17 | H2-IAd | 129 | 143 | KGPAAVAVDVESDFL | 1.0037 |
