# Supplementary material for: Development of multi-epitope Cathepsin L driven short peptide vaccine against Fasciola gigantica
Source: Front Vet Sci. 2025 May 22;12:1547937. doi: 10.3389/fvets.2025.1547937 (PMC12139528; doi:10.3389/fvets.2025.1547937)
Supplement: Supplementary file 3 [file Supplementary_Table_2.DOCX]

Supplementary Material

**Supplementary Table 2.** The percent conserved residues of overlapped BCL and HTL groups.

| **Group** | **Conserved residues (aa)** | **Total residue (aa)** | **Percent conserved residues** |
| --- | --- | --- | --- |
| B1 | 9 | 15 | 60 |
| B2 | 3 | 6 | 50 |
| T1 | 11 | 20 | 55 |
